# Gender Differences in the Recognition of Vocal Emotions

**DOI:** 10.3389/fpsyg.2018.00882

**Published:** 2018-06-05

**Authors:** Adi Lausen, Annekathrin Schacht

**Affiliations:** ^1^Department of Affective Neuroscience and Psychophysiology, Institute for Psychology, University of Goettingen, Goettingen, Germany; ^2^Leibniz Science “Primate Cognition”, Goettingen, Germany

**Keywords:** gender differences, emotion recognition accuracy, voice, speech-embedded emotions, affect bursts

## Abstract

The conflicting findings from the few studies conducted with regard to gender differences in the recognition of vocal expressions of emotion have left the exact nature of these differences unclear. Several investigators have argued that a comprehensive understanding of gender differences in vocal emotion recognition can only be achieved by replicating these studies while accounting for influential factors such as stimulus type, gender-balanced samples, number of encoders, decoders, and emotional categories. This study aimed to account for these factors by investigating whether emotion recognition from vocal expressions differs as a function of both listeners' and speakers' gender. A total of *N* = 290 participants were randomly and equally allocated to two groups. One group listened to words and pseudo-words, while the other group listened to sentences and affect bursts. Participants were asked to categorize the stimuli with respect to the expressed emotions in a fixed-choice response format. Overall, females were more accurate than males when decoding vocal emotions, however, when testing for specific emotions these differences were small in magnitude. Speakers' gender had a significant impact on how listeners' judged emotions from the voice. The group listening to words and pseudo-words had higher identification rates for emotions spoken by male than by female actors, whereas in the group listening to sentences and affect bursts the identification rates were higher when emotions were uttered by female than male actors. The mixed pattern for emotion-specific effects, however, indicates that, in the vocal channel, the reliability of emotion judgments is not systematically influenced by speakers' gender and the related stereotypes of emotional expressivity. Together, these results extend previous findings by showing effects of listeners' and speakers' gender on the recognition of vocal emotions. They stress the importance of distinguishing these factors to explain recognition ability in the processing of emotional prosody.

## Introduction

The ability to accurately perceive the emotional states of others is a fundamental socio-cognitive ability for the successful regulation of our interpersonal relationships (Levenson and Ruef, 1992; Fischer and Manstead, 2008) and it relies on the integration of several information cues such as facial expressions, tone of voice (prosody), words or body language (Van den Stock et al., 2007; Jessen and Kotz, 2011). Although there is a consensus among researchers that the recognition of emotions is facilitated by the availability of additional sensory channels (de Gelder and Vroomen, 2000; Paulmann and Pell, 2010; Klasen et al., 2014), it has also been shown that using just one channel (e.g., the voice) is more than sufficient at deciphering a person's emotional state well above chance (Apple and Hecht, 1982; Juslin and Laukka, 2001; Thompson and Balkwill, 2006, 2009; Jürgens et al., 2018).

The voice is a highly complex tool of communication or, as already Darwin (1872/1998) pointed out, the most indicative of an individual's emotional state. Our voice discloses information not only about our biological, psychological or social status (e.g., Azul, 2013) but also expresses emotions using different domains such as prosody, semantics or non-speech sounds (i.e., affect bursts; e.g., Schwartz and Pell, 2012; Kraus, 2017). Several studies have demonstrated that the main and most obvious function of prosody and non-speech sounds is that of facilitating interaction and communication (see for example, Belin, 2006; Hawk et al., 2009; Belin et al., 2011; Paulmann et al., 2012; Pell et al., 2015; Fischer and Price, 2017, for details). One of the methodological challenges when studying prosody in human speech is how to isolate processes related to the *encoding* (expressing) and *decoding* (judging) of emotions from those of processing semantic information carried by, for example, words or sentences. To circumvent this problem, researchers used either pseudo-speech or affect bursts (e.g., simulated laughter, crying) as stimulus material. While the former captures the pure effects of emotional prosody independent of lexical-semantic cues, the latter has been argued to have an adaptive value (Fischer and Price, 2017) and to be an ideal tool when investigating the expression of emotional information when there is no concurrent verbal information present (Pell et al., 2015).

In the context of nonverbal communication (e.g., vocal affect, facial expressions, body language), gender has been repeatedly proposed as an important factor that might influence the accuracy of performance in emotion recognition tasks (e.g., Hall, 1978, 2006; Hall et al., 2000; Sokolov et al., 2011; Fischer and Evers, 2013; Thompson and Voyer, 2014; Santos and Osório, 2015). One can distinguish two major lines of research. One line assumes that females and males differ in their emotionality, personality, abilities, attitudes or behavioral tendencies (*gender differences hypothesis*; Gray, 1992) and that women are “emotional experts”, more inclined to pay attention to their own and others' feelings and intuitions (Hess et al., 2000; Shields, 2002; Timmers et al., 2003). Several studies have shown that both genders differ in the way they express (e.g., Barett and Bliss-Moreau, 2009; Parkins, 2012; McDuff et al., 2017), experience (e.g., Šolcová and Lacev, 2017), and decode or encode emotions with females outperforming males when completing tasks designed to measure non-verbal communication ability (e.g., Zuckerman et al., 1975; Ambady and Rosenthal, 1998; MacAdams, 2012; Wells et al., 2016; Wingenbach et al., 2018). In addition, meta-analytic reviews, summarizing work on gender differences concerning the ability to recognize non-verbal expressions of emotion, also reported a female advantage for emotion recognition tasks with effect sizes ranging from small to medium (e.g., Hall, 1984; McClure, 2000). Explanations for these gender-based behavior patterns range from socio-cultural influences and psychological dispositions to evolutionary perspectives (see Briton and Hall, 1995; Brody, 1997; Eagly and Wood, 1999; Davis et al., 2012; for more detailed explanations). For instance, it has been suggested that females, due to their responsibility for child rearing, are expected to be prosocial and nurturing and, thus, more responsive and accurate in judging other people's emotions (Hall, 1984; Babchuk et al., 1985; Schirmer, 2013).

Conversely, the other line of research has emphasized the homogeneity between genders across various domains (e.g., non-verbal communication, social and personality variables, psychological well-being) based on evidence from meta-analyses. For instance, Richard et al. (2003) examined gender differences across domains by using a second *order meta-analysis* (see Schmidt and Oh, 2013; Zell and Krizan, 2014, for details) to characterize the average difference between males and females. With regard to nonverbal communication the authors aggregated the data from a series of experiments conducted by Rosenthal and DePaulo (1979) and found that the correlation coefficients between genders were small, ranging from *r* = 0.16, for facial cues, *r* = 0.11, for body cues to *r* = 0.06, for vocal cues. Furthermore, Hyde (2005, 2014) observed 78% of effect sizes to be small or close to zero, leading her to conclude that in many cases females and males are rather similar on most psychological dimensions (*gender similarity hypothesis*). The results of these meta-analytic reviews are useful for estimating the overall magnitude and variability of female-male comparisons across various domains. However, this line of research might under-interpret the differences between females and males for emotion recognition by failing to consider modality specific effects (Abelson, 1985; Thompson and Voyer, 2014). A comprehensive conclusion cannot be drawn when the vast majority of evidence comes from studies that assess gender effects mainly within only one modality (e.g., Hyde, 2005) or by employing only one test (*Profile of Nonverbal Sensitivity*, Rosenthal and DePaulo, 1979) to assess performance accuracy for decoding nonverbal cues (e.g., Richard et al., 2003). Thus, until further evidence on the similarities and differences between genders within specific sensory modalities is provided, the direction of these effects remains an open question.

Contrary to the growing field of research examining gender effects in the recognition of emotions within the visual modality, where researchers are working toward improving methodology by either including facial expressions with varying intensity (Wingenbach et al., 2018), dynamically rising expressions (e.g., Recio et al., 2011, 2014), or different stimulus types such as avatars, human faces or icons (Fischer et al., 2018), the investigation of these effects within the vocal domain is still understudied. This paucity persists despite a common consensus that the voice is an important source of social information (e.g., Latinus and Belin, 2011; Morningstar, 2017). Research comparing auditory, visual, and audio-visual modalities reported significant main effects of gender (Scherer and Scherer, 2011), with females outperforming males in all three conditions of stimulus presentation (Collignon et al., 2010). Similarly, Lambrecht et al. (2014) demonstrated a significant female advantage in emotion recognition which was however restricted to vocal emotions. A female advantage was also found in studies investigating emotion recognition purely within the vocal domain (e.g., Scherer et al., 2001; Toivanen et al., 2005; Paulmann and Uskul, 2014; Demenescu et al., 2015). These findings were corroborated by Keshtiari and Kuhlmann (2016), who investigated how gender affects the recognition of vocal expressions of emotion. Participants listened to sentences spoken in five different emotions (angry, disgust, fear, happiness, and sadness) or in a neutral tone of voice and made a decision on the emotional category the presented utterances corresponded to. Results revealed a significant main effect of gender with an overall recognition advantage for females, confirming in this way the consistency of findings in past research. Other studies, however, reported either only a small overall advantage in favor of females in the recognition of non-verbal (auditory, visual, audio-visual) displays of emotion (Kret and de Gelder, 2012; Thompson and Voyer, 2014) or even equal performance accuracy for male and female participants in identifying emotions from both, speech-embedded (e.g., Raithel and Hielscher-Fastabend, 2004; Paulmann et al., 2008; Sauter et al., 2013) and non-speech sounds (e.g., Hawk et al., 2009; Lima et al., 2014).

To address these diverging findings, it has been suggested that instead of examining gender effects across emotions, specific emotion categories should be considered separately (de Gelder, 2016). For instance, in a behavioral study Bonebright et al. (1996) examined participants' ability to decode emotions from vocal cues. They instructed trained actors to record paragraph-long stories, each time using their voice to portray a specified emotion (i.e., anger, fear, happiness, sadness, and neutral). Subsequently, undergraduate students listened to each recorded paragraph and tried to determine which emotion the speaker was trying to portray. Females were significantly more accurate than males in decoding voices that expressed fear, happiness, and sadness. These gender differences were small but consistent. No gender differences were found for emotional expressions uttered in an angry or neutral tone of voice. Subsequent evidence showed that females outperform males for utterances spoken in a *fearful* (Demenescu et al., 2015; Zupan et al., 2016), *happy* (Fujisawa and Shinohara, 2011; Lambrecht et al., 2014; Demenescu et al., 2015; Zupan et al., 2016), and *sad* (Fujisawa and Shinohara, 2011; Zupan et al., 2016) tone of voice. While both genders were found to perform equally well when identifying *angry* (Fujisawa and Shinohara, 2011; Lambrecht et al., 2014; Demenescu et al., 2015; Zupan et al., 2016), and *neutral* (Demenescu et al., 2015) prosody, other investigators failed to replicate these findings and found higher accuracy for females in correctly recognizing neutral vocalizations (Lambrecht et al., 2014), or no gender differences in the recognition of sad prosody (Demenescu et al., 2015). That the accuracy of performance varies across discrete emotion categories (e.g., fear, sadness or happiness was argued to play a greater role in women, whereas anger and disgust in men) might be the result of biological or environmental factors, which are likely to trigger “qualitatively” different emotional experiences for men and women (see Schirmer, 2013, for a comprehensive review).

The above-mentioned studies do not show a consistent gender pattern either regarding overall effects in the performance accuracy of decoding vocal emotions or emotion specific categories [see Table 1 (a1) for overall effects in decoding vocal emotions and (a2) for decoding performance accuracy by emotion categories]. There are several likely sources for these inconsistencies. One of the reasons may have been the large variety of different types of vocal stimuli (e.g., words, pseudo-words, sentences, pseudo-sentences, affect bursts). Other methodological differences that might have been responsible for these conflicting results are related either to the *number of emotions* studied [which vary from two (e.g., Collignon et al., 2010) to nine (e.g., Belin et al., 2008)], the *language under investigation* (e.g., Scherer et al., 2001; Keshtiari and Kulhmann, 2016), the *population in question* [children (e.g., Fujisawa and Shinohara, 2011; Sauter et al., 2013), young adults (e.g., Scherer et al., 2001; Paulmann and Uskul, 2014), older adults (e.g., Lima et al., 2014), clinical populations (e.g., Zupan et al., 2016)], *unbalanced gender groups* [e.g., 71F/50M (Hawk et al., 2009)], and the *sample size* [which range from 24 (e.g., Raithel and Hielscher-Fastabend, 2004) to 428 (e.g., Scherer et al., 2001)]. The gender of the actor/actress portraying different emotions is a further variable of interest that has been proposed to influence the overall performance accuracy when identifying emotions from the voice (e.g., Scherer et al., 2001). In a validation study concerning the identification of vocal emotions, Belin et al. (2008) tested for differences in performance accuracy based on listeners' as well as speakers' gender. Participants were asked to evaluate actors' vocalizations on three emotional dimensions: valence, arousal, and intensity. Results showed higher mean identification rates (for intensity and arousal dimensions) across all emotion categories when spoken by female actors. Similar to other findings (e.g., Bonebright et al., 1996; Lambrecht et al., 2014), Belin et al. (2008) found no significant interaction between listeners' gender, speakers' gender and emotions, but a significant main effect for listeners' and speakers' gender. These findings indicate that females compared to males were not only better at decoding but also at identifying emotions in the female voice. Considering emotion-specific effects, it has been shown that vocal portrayals of anger and fear have higher mean identification rates when spoken by male actors (Bonebright et al., 1996), whereas happy (Bonebright et al., 1996), and neutral expressions (Young et al., 2017) were better identified from female voices. In contrast, other investigators observed that fear and disgust were better identified when spoken by a female (though a response bias toward disgust when an actor portrayed the emotion and, fear when an actress expressed the emotion was reported; see Collignon et al., 2010, for details). Further research that includes speakers' gender as an additional factor, reports that while gender differences might exist for identifying emotions from speakers' voice, these are not systematic and vary for specific emotions (Pell et al., 2005; Hawk et al., 2009) or occur regardless of the actors' gender (Schirmer and Kotz, 2003; Riviello and Esposito, 2016). Similar to the performance accuracy of decoding emotions, the evidence with regard to speaker's gender as a relevant factor for identifying emotions from the voice is inconsistent [see Table 1 (b1) for overall identification rates by speakers' gender and (b2) for identification rates by speakers' gender and emotion category]. The discrepancies in these findings are likely to be attributable to a number of methodological differences, such as *recording conditions* (e.g., Burkhardt et al., 2005), *number of speakers* which vary from 2 (e.g., Demenescu et al., 2015) to 14 (Toivanen et al., 2005) or validity of prosodic stimuli derived from the *simulation of emotional expressions* (see Hawk et al., 2009; Jürgens et al., 2015, for a discussion whether authentic vs. play acted emotional speech may lower ecological validity).

Table 1Gender differences: main findings of previous studies.

**Table:** 

**Studies**	**Stimulus types**	**(a1) Overall effects of decoding vocal emotions**	
		**Female (F)**	**Male (M)**
Bonebright et al., 1996	Short stories	↑	↓
Scherer et al., 2001; Paulmann and Uskul, 2014	Pseudo-sentences	↑	↓
Belin et al., 2008; Collignon et al., 2010	Affect bursts	↑	↓
Demenescu et al., 2015	Pseudo-words	↑	↓
Toivanen et al., 2005; Keshtiari and Kuhlmann, 2016; Zupan et al., 2016	Lexical and neutral sentences	↑	↓
Hawk et al., 2009; Sauter et al., 2013; Lima et al., 2014	Affect bursts and three-digit numbers	*n.s*.	
Raithel and Hielscher-Fastabend, 2004; Paulmann et al., 2008	Lexical and neutral sentences	*n.s*.

**Table:** 

		**(a2) Decoding accuracy by emotion category**													
		***Ha***		***An***		***Di***		***Fe***		***Sa***		***Su***		***Ne***	
		**F**	**M**	**F**	**M**	**F**	**M**	**F**	**M**	**F**	**M**	**F**	**M**	**F**	**M**
Bonebright et al., 1996	Short stories	↑	↓	*n.s*.				↑	↓	↑	↓			*n.s*.	
Fujisawa and Shinohara, 2011	Words	↑	↓	*n.s*.						↑	↓			*n.r*.	
Lambrecht et al., 2014	Words	↑	↓	*n.s*.		*n.s*.								↑	↓
Demenescu et al., 2015	Pseudo-words	↑	↓	*n.s*.		*n.s*.		↑	↓	*n.s*.				*n.s*.	
Zupan et al., 2016	Neutral sentences and short stories	*n.s*.		*n.s*.				↑	↓	↑	↓

**Table:** 

		**(b1) Overall identification rates of vocal emotions by the gender of encoder**	
		**Female (F)**	**Male (M)**
Scherer et al., 2001	Pseudo-sentences	↑	↓
Belin et al., 2008; Collignon et al., 2010	Affect bursts	↑	↓
Riviello and Esposito, 2016	Audio clips	*n.s*.	
Lambrecht et al., 2014	Words	*n.s*.

**Table:** 

		**(b2) Identification rates by encoders' gender and emotion category**													
		***Ha***		***An***		***Di***		***Fe***		***Sa***		***Su***		***Ne***	
		**F**	**M**	**F**	**M**	**F**	**M**	**F**	**M**	**F**	**M**	**F**	**M**	**F**	**M**
Bonebright et al., 1996	Short stories	↑	↓	↑	↓			↑	↓	*n.s*.				*n.r*.	
	Pseudo-sentences (German)	*n.s*.		*n.s*.		↓	↑	*n.s*.		↑	↓	*n.s*.		*n.s*.	
Pell et al., 2005	Pseudo-sentences (English)	↑	↓	↓	↑	↓	↑	*n.s*.		*n.s*.		↑	↓	*n.s*.	
	Pseudo-sentences (Arabic)	*n.s*.		↑	↓	*n.s*.		*n.s*.		*n.s*.		*n.s*.		*n.s*.	
Collignon et al., 2010	Affect bursts					↑	↓	↑	↓						

*Ha, Happy; An, Angry; Di, Disgust; Fe, Fear; Sa, Sad; Su, Surprise; Ne, Neutral. The shades indicate the absence of emotions; n.s., not significant; n.r., not reported;↑/↓, better/lower performance of decoding vocal emotions by listeners' gender (**a1**) and emotion category (**a2**); better/lower performance of identifying vocal emotions by speakers' gender (**b1**) and emotion category (**b2**)*.

A seemingly inevitable conclusion after reviewing past work on gender differences in the recognition of vocal expressions of emotion is that conflicting findings have left the exact nature of these differences unclear. Although accuracy scores from some prior studies suggest that females are overall better than males at decoding and encoding vocal emotions, independent of the stimulus type, other studies do not confirm these findings. Likewise, the question whether women are consistently better than men at decoding and identifying emotions such as happiness, fear, sadness or neutral expressions when spoken by a female, while men have an advantage for anger and disgust, remains unresolved. The absence of consistent gender effects for the encoding and decoding of emotional vocal expressions might be a result of the selected stimuli, either speech-embedded (pseudo/words, pseudo/sentences) or non-verbal vocalizations (affect bursts). Thus, it has been suggested that a comprehensive understanding of gender differences in vocal emotion recognition can only be achieved by replicating these studies while accounting for influential factors such as stimulus type, gender-balanced samples, number of encoders, decoders, and emotional categories (Bonebright et al., 1996; Pell, 2002; Lambrecht et al., 2014; Ba̧k, 2016).

To address some of these limitations, the present study aimed at investigating, across a large set of speech-embedded stimuli (i.e., words, pseudo-words, sentences, pseudo-sentences) and non-verbal vocalizations (i.e., affect bursts) whether emotion recognition of vocal expressions differs as a function of both decoders' and encoders' gender and to provide parameter estimates on the magnitude and direction of these effects. To date, no extensive research on differences between males and females in the recognition of emotional prosody has been conducted and, thus, we based our approach for investigating these effects on the patterns observed in the majority of the aforementioned studies. We first examined whether there are any differences in the performance accuracy of decoding vocal emotions based on listeners' gender (i.e., across all stimuli and for each stimulus type; across all emotions and for each emotion category). Specifically, we expected an overall female advantage when decoding vocal emotions, and that they would be more accurate than males when categorizing specific emotions such as *happiness, fear, sadness*, or *neutral* expressions. No gender differences were expected to manifest for emotions uttered in an *angry* and *disgusted* tone of voice. Secondly, we tested whether there are any differences for identifying vocal emotions based on speakers' gender (i.e., across all stimuli and for each stimulus type; across all emotions and for each emotion category). We hypothesized that vocal portrayals of emotion would have overall significantly higher hit rates when spoken by female than by male actors. Considering emotion-specific effects, we expected that *anger* and *disgust* would have higher identification rates when spoken by male actors, whereas portrayals of *happiness, fear, sadness*, and *neutral* would be better identified when spoken by female actors. Finally, we investigated potential interactions between listeners' and speakers' gender for the identification of vocal emotions across all stimuli and for each stimulus type.

## Methods

The study was conducted in accordance with the ethical principles formulated in the *Declaration of Helsinki* and approved by the ethics committee of the *Georg-Elias-Müller- Institute of Psychology*, University of Goettingen, Germany.

## Participants

Participants were *N* = 302 volunteers (age range 18–36) from the University of Goettingen and the local community. They were recruited through flyers distributed at the University campus, the *ORSEE* database for psychological experiments (http://www.orsee.org/web/), postings on the social media site *Facebook* and the online platform *Schwarzes Brett* Goettingen (https://www.uni-goettingen.de/en/644.html). Inclusion criteria for participation in the study were: native speakers of German, aged above eighteen, normal hearing, not currently taking medication affecting the brain and no self-reported mental health problems. Twelve participants who reported hearing disorders (e.g., tinnitus), psychiatric/neurological disorders or the intake of psychotropic medication were not eligible to participate. This left a total of 290 participants (143 female, 147 male) with a mean age of 23.83 years (*SD* = 3.73). To assess the performance accuracy between females and males within different types of vocal stimuli (i.e., words, pseudo-words, sentences, pseudo-sentences, affect bursts) and to reduce the length of the experiment participants were randomly allocated to two groups of equal size. This allowed us to have a higher number of stimuli in each group resulting in a higher precision of estimated gender or emotion differences within one database and respectively within one of the groups. One group classified words and pseudo-words stimuli (*n* = 145, *M*_*age*_ = 24.00, *SD* = 3.67), whereas the other group was presented with stimuli featuring sentences, pseudo-sentences, and affect bursts (*n* = 145, *M*_*age*_ = 23.66, *SD* = 3.80). To assess whether there were any age differences in the two groups a *Wilcoxon-Mann-Whitney test* was conducted. The results indicated a significant age difference between females and males in both groups (*Group*_*Words*_: *z* = −2.91, *p* = 0.004; *Group*_*Sentences*_: *z* = −2.79, *p* = 0.005). Participants' demographic characteristics are presented in Table 2.

**Table 2:** Demographic characteristics of the study population.

**Group**	**Gender**		**Age**	**Education**			
		***n***	***M* (*SD*)**	***HS-Dipl*.**	***A-levels***	***BA***	***MA***
Words	Females	71	23.10 (3.31)	1	46	21	3
	Males	74	24.86 (3.80)	1	45	20	8
Sentences	Females	72	22.72 (3.29)		46	20	6
	Males	73	24.57 (4.06)		44	12	17

*HS-Dipl, Highschool diploma (i.e., Realschulabschluss); BA, Bachelor; MA, Master*.

Throughout the article these two groups will be referred to as *Group-Words* and *Group-Sentences*. Participants were reimbursed with course credit or 8 Euros.

### Materials and stimuli selection

The speech/non-speech embedded stimuli were extracted from well-established and validated databases or provided by researchers who developed their own stimulus materials [see Table 3 for a brief description on the features of the selected databases (e.g., stimuli types, number of speakers)].

**Table 3:** Features of the selected emotion speech databases^a^.

**Database**	**Speakers**	**Emotions**	**Nature of material**	**Total stimuli**
*Anna* (Hammerschmidt and Jürgens, 2007)	22 drama students (10 male/12 female)	Anger, affection, contempt, despair, fear, happiness, sensual satisfaction, triumph, neutral	Word	*N*_Stimuli_ = 198
*Berlin Database of Emotional Speech* (EMO_DB) (Burkhardt et al., 2005)	10 untrained actors (5 male/5 female)	Anger, boredom, disgust, fear, happiness, sadness, neutral	Semantic neutral sentences	*N*_Stimuli_ = 816
*Magdeburg Prosody Corpus* (WASEP) (Wendt and Scheich, 2002)	2 actors (1 male/1female)	Anger, disgust, fear, happiness, sadness, neutral	Pseudo-words Nouns^b^	*N*_Stimuli_ = 222 *N*_Stimuli_ = 3,318
*Montreal Affective Voices* (MAV) (Belin et al., 2008)	10 actors (5 male/5 female)	Anger, disgust, fear, happiness, pain, pleasure, sadness, surprise, neutral	Affect bursts	*N*_Stimuli_ = 90
*Paulmann Prosodic Stimuli* (Paulmann and Kotz, 2008; Paulmann et al., 2008)	2 actors (1 male/1female)	Anger, disgust, fear, happiness, sadness, surprise, neutral	Pseudo- sentences Lexical sentences^c^	*N*_Stimuli_ = 210 *N*_Stimuli_ = 210

a *The word databases it is used as a generic term as some of the selected stimuli are from researchers that developed their own stimulus materials with no aim of establishing a database (i.e., Anna and Paulmann prosodic stimuli)*.

b *The nouns from WASEP are classified according to their positive, negative and neutral semantic content*.

c *Paulmann lexical sentences consists of semantically and prosodically matching stimuli. Compared to all other types of stimuli, which were cross-over designed (i.e., stimulus is spoken in all emotional categories) both, the pseudo- and lexical sentences from Paulmann et al. (2008) database were hierarchically designed (i.e., stimulus is spoken only in one emotional category). The validation procedures of the stimuli are presented in the above-cited papers*.

To be included in the present study the stimuli had to satisfy the following criteria: (1) be spoken in a neutral tone (i.e., baseline expression) or in one of the emotion categories of interest (i.e., happiness, surprise, anger, fear, sadness, disgust), (2) to be recorded under standardized conditions, (3) to have at least two encoders (i.e., male/female), and (4) to be produced by human expressers.

We decided to use a wide variety of stimuli representing the spectrum of materials used in emotional prosody research (i.e., for speech: words, lexical and neutral sentences; pseudo-speech: pseudo-words/sentences; for non-speech: vocalizations). For economic reasons, only a sub-set of stimuli from each database was selected. For *Anna* and *Montreal Affective Voices* (*MAV*) databases all speakers for the emotion category of interest were chosen. This resulted in a total number of 88 Stimuli for *Anna* [4 Emotions (anger, happiness, sadness, neutral) × 22 Speakers] and 70 Stimuli for *MAV* [7 Emotions (anger, disgust, fear, happiness, sadness, surprise, neutral) × 10 Speakers]. The stimuli from the remaining other three databases were ordered randomly and the first 10 items per database were selected. Stimulus selection resulted in a total number of 280 stimuli from the *Paulmann Prosodic Stimuli* set [10 *Pseudo-sentences* × 7 Emotions (anger, disgust, fear, happiness, sadness, surprise, and neutral) × 2 Speakers; 10 *Lexical Sentences* × 7 Emotions (anger, disgust, fear, happiness, sadness, surprise, and neutral) × 2 Speakers], 120 stimuli from the *Berlin Database of Emotional Speech* [10 *Semantic Neutral Sentences* × 6 Emotions (anger, disgust, fear, happiness, sadness, and neutral) × 2 Speakers] and 480 Stimuli from the *Magdeburg Prosody Corpus* [10 *Pseudo-words* × 6 Emotions (anger, disgust, fear, happiness, sadness, and neutral) × 2 Speakers; 10 *Semantic positive nouns*/10 *Semantic negative nouns*/10 *Semantic neutral nouns* × 6 Emotions (anger, disgust, fear, happiness, sadness, and neutral) × 2 Speakers]. The nouns extracted from the *Magdeburg Prosody Corpus* were additionally controlled for valence, arousal, and word frequency according to the *Berlin Affective Word List Reloaded* (Võ et al., 2009).

### Acoustic analysis

The extraction of amplitude (*dB*), duration, and peak amplitude of all 1,038 original stimuli was conducted using the phonetic-software *Praat* (Boersma, 2001). As the stimuli used for this study came from different databases with different recording conditions, we controlled for acoustic parameters, including the minimum, maximum, mean, variance, and standard deviation of the amplitude. The results of our analyses indicated that the variation coefficient (*C*_*V*_) for amplitude between the stimuli was high (*s*^*2*^ = 71.92, *M* = 63.06, *C*_*V*_ = 13.45%). Therefore, the stimuli were normalized with regards to loudness by applying the *Group Waveform Normalization* algorithm of *Adobe Audition CC* (Version 8.1, Adobe Systems, 2015, San Jose, CA) that uniformly matches the loudness based on the *root-mean-square* (RMS) levels. To control whether normalization worked, the stimuli were re-uploaded in *Praat*, which indicated that the variation coefficient between the stimuli was reduced by roughly 40% (*s*^*2*^ = 24.97, *M* = 61.07, *C*_*V*_ = 8.18%) by this procedure.

Physical volume of stimulus presentation across the four PCs' used in the experiment was controlled by measuring sound volume of the practice trials with a professional sound level meter, *Nor140* (Norsonic, 2010, Lierskogen, Norway). No significant difference in volume intensity was observed [*F*_(3, 27)_ = 0.53, *p* = 0.668].

### Procedure

Participants were tested in groups of up to four members. At arrival, each participant was seated in front of a *Dell OptiPlex*™ *780* Desktop-PC. All participants were provided with individual headphone devices (*Bayerdynamic DT 770 PRO*). After signing a consent form and completing a short demographic questionnaire concerning age, gender^1^ and education level, participants were informed that they would be involved in a study evaluating emotional aspects of vocal stimulus materials. Afterwards, they were told to put on headphones and carefully read the instructions presented on the computer screen. Before the main experiment, participants were familiarized with the experimental setting in a short training session comprised of 10 stimuli, which were not presented in the main experiment. They were instructed to carefully listen to the presented stimuli as they would be played only once and that the number of emotions presented might vary form the number of categories given as possible choices (see Design and Randomization for an argument related to this approach). Each trial began with a white fixation-cross presented on a gray screen, which was shown until participants' response had been recorded. The presentation of the stimuli was initiated by pressing the *Enter*-key. After stimulus presentation, participants had to decide as accurately as possible, in a fixed-choice response format, which of the 7 emotional categories (i.e., anger, disgust, fear, happiness, sadness, surprise, neutral) the emotional prosody of the presented stimulus corresponded to. Following their emotion judgment, they were asked the correctness of their answer on a 7-point Likert scale, where “1” corresponded to *not at all confident* and “7” corresponded to *extremely confident*. The responses were made using the marked computer keyboard (*Z* to *M* for the emotion judgments, which were labeled corresponding to the emotion categories, and *1* to *7* for confidence). There was no time limit for emotion judgments or confidence ratings. At the end of each block a visual message in the center of the screen instructed participants to take a break if they wished to or to press the *Spacebar* to proceed with the next block. The 568 stimuli for *Group Words* had a mean duration of 1.03 ± 0.36 s, whereas in Group Sentences the mean duration of the 470 stimuli was 2.66 ± 1.01 s. Testing took approximately 60 min for both groups.

### Design and randomization

We fitted a balanced design to allow for a separate analysis of effects across the recognizability of emotional expressions, skill in judging emotional expressions, and the interaction between encoding and decoding (the assumptions of such an approach were justified by Elfenbein and Ambady, 2002a,b). Following the argumentation of Wagner (1993), participants were provided with the same number of judgment categories, independent of the given emotion categories within the included databases. This approach guarantees that, the response probabilities are not influenced by the different number of emotional categories (i.e., the probability of correct/false recognition of emotions by random choice is equal).

The set of stimuli for the *Group Words* was split into three blocks (*Anna, Pseudo-words*, and *Nouns*) while the set of stimuli for the *Group Sentences* was split into four blocks (*Pseudo-sentences, Lexical Sentences, Neutral sentences*, and *Affect bursts*). Each block as well as the stimuli within each block were randomized using the software *Presentation* (Version 14.1, Neurobehavioral Systems Inc., Albany, CA).

### Sample size calculations

A target sample size of 134 participants per group (67F/67M) was determined using *Wilcoxon Mann-Whitney* two-tailed test (*d* = 0.50; α = 0.05; *1–*β = 0.80). Assuming 67 participants in each gender group and the minimum number of observations per participant (i.e., 70) we further investigated, via a two-sample binomial test, whether the determined sample size possessed enough statistical power to assess the size of females'/males' differences in detecting vocal emotions. This argument indicated that at 80% recognition probability the sample size was powered enough to detect small differences as 2.3%. To take account of possible attrition the sample size was increased by at least 10%.

### Statistical analysis

The data was analyzed by a *generalized linear model* (*quasi-binomial logistic regression*) for the binary response variable emotion recognition. As individual effects (e.g., fatigue, boredom) might impact cognitive performance, we treated *participants* as a confounder in our model. In addition, we controlled for *confidence*, as a confounder, shown to impact on performance accuracy in emotion recognition tasks (e.g., Beaupré and Hess, 2006; Rigoulot et al., 2013). Our analysis on the baseline characteristics of the study population indicated a significant age effect between males and females and, therefore, we additionally included this factor as a confounder in our model. Listeners' gender, speakers' gender, emotions and stimulus type were included as predictor variables. Age was included as a quantitative variable. Listeners' gender and speakers' gender were included as binary variables and confidence, participant, emotion, and stimulus type were included as nominal variables. The dispersion parameter of the quasi-binomial model and the nominal variable participants accounted for dependencies caused by repeated measurements within the participants. First order interactions were fitted between *listeners' gender* and *speakers' gender, age* and *stimuli types, confidence* and *stimuli types, speakers' gender* and *stimuli types, listeners' gender* and *stimuli types, emotions* and *stimuli types, age* and *speakers' gender, age* and *emotions, listeners' gender* and *emotions, speakers' gender* and *emotions, confidence* and *participant*. A second order interaction was fitted between *listeners' gender, speakers' gender*, and *emotions*. Chi-square tests of the deviance analysis (generalized mixed model analysis) were used to analyze additive and interaction effects.

Means, standard deviations, z-scores, p-values and effect sizes were calculated to describe the differences between genders in performance accuracy. This descriptive analysis was conducted using the unadjusted group means, which allows the application of non-parametric robust methods, direct illustration and interpretation of the effect sizes and patterns. As emotion recognition is binomial distributed and does not allow the assumption of a normal distribution we used *Wilcoxon-Mann-Whitney test* for independent samples to analyze the effects of listeners' gender and *Wilcoxon-rank-sum test* for dependent samples to analyze the effects of speakers' gender. Corrections for multiple testing were implemented using Bonferroni's method for multiple comparisons.

The data was analyzed using the R language and environment for statistical computing and graphics version 3.3.1 (R Core Team, 2016) and the integrated environment R-Studio version 1.0.316. The quasi-binomial logistic regression was fitted using the R function glm. *Wilcoxon-Mann-Whitney* and *Wilcoxon-rank-sum* were performed with the R package *coin* introduced by Hothorn et al. (2008).

## Results

### Emotion effects on performance accuracy

The quasi-binomial logistic models revealed significant interactions between emotions and stimuli types in both groups [*Group Words*: $\chi_{\left(18\right)}^{\text{2}}$ = 1097.80, *p* < 0.001; *Group Sentences*: $\chi_{\left(17\right)}^{\text{2}}$ = 1990.40, *p* < 0.001]. Main effects of emotion were observed across all stimuli [*Group Words*: $\chi_{\left(5\right)}^{\text{2}}$ = 4853.80, *p* < 0.001; *Group Sentences*: $\chi_{\left(6\right)}^{\text{2}}$ = 6956.00, *p* < 0.001] and for each stimulus type [*Anna*: $\chi_{\left(3\right)}^{\text{2}}$ = 2463.87, *p* < 0.001; *pseudo-words*: $\chi_{\left(5\right)}^{\text{2}}$ = 1060.19, *p* < 0.001; *semantic positive nouns*: $\chi_{\left(5\right)}^{\text{2}}$ = 616.96, *p* < 0.001; *semantic negative nouns*: $\chi_{\left(5\right)}^{\text{2}}$ = 735.54, *p* < 0.001; *semantic neutral nouns*: $\chi_{\left(5\right)}^{\text{2}}$ = 1603.56, *p* < 0.001; *pseudo-sentences*: $\chi_{\left(6\right)}^{\text{2}}$ = 2784.06, *p* < 0.001; *lexical sentences*: $\chi_{\left(6\right)}^{\text{2}}$ = 3745.60, *p* < 0.001; *neutral sentences*: $\chi_{\left(5\right)}^{\text{2}}$ = 1332.93, *p* < 0.001; and *affect bursts*: $\chi_{\left(6\right)}^{\text{2}}$ = 1113.20, *p* < 0.001]. The full models across all databases and for each stimulus type are presented in Supplementary Material (see Tables S1a,b, S2a–i).

### Decoding performance accuracy by listeners' gender

Significant first order interactions between listeners' gender and stimuli types were observed for both groups [*Group Words*: $\chi_{\left(4\right)}^{\text{2}}$ = 16.40, *p* = 0.038; *Group Sentences*: $\chi_{\left(3\right)}^{\text{2}}$ = 22.80, *p* < 0.001]. A significant main effect of gender was found across the stimuli types for *Group Words* [$\chi_{\left(1\right)}^{\text{2}}$ = 51.70, *p* < 0.001] but not for *Group Sentences* [$\chi_{\left(1\right)}^{\text{2}}$ = 5.20, *p* = 0.332]. Main effects of gender were revealed for the following stimulus sub-sets: *pseudo-words* [$\chi_{\left(1\right)}^{\text{2}}$ = 29.18, *p* < 0.001], *semantic positive nouns* [$\chi_{\left(1\right)}^{\text{2}}$ = 20.14, *p* < 0.001], *semantic negative nouns* [$\chi_{\left(1\right)}^{\text{2}}$ = 8.38, *p* = 0.046] and *semantic neutral nouns* [$\chi_{\left(1\right)}^{\text{2}}$ = 9.13, *p* = 0.029]. No main effects of gender were found for *Anna* [$\chi_{\left(1\right)}^{\text{2}}$ = 0.03, *p* = 1.00], *pseudo-sentences* [$\chi_{\left(1\right)}^{\text{2}}$ = 7.44, *p* = 0.068], *lexical sentences* [$\chi_{\left(1\right)}^{\text{2}}$ = 5.50, *p* = 0.211], *neutral sentences* [$\chi_{\left(1\right)}^{\text{2}}$ = 5.35, *p* < 0.243] and *affect bursts* [$\chi_{\left(1\right)}^{\text{2}}$ = 2.83, *p* = 0.698].

**Figure 1:**
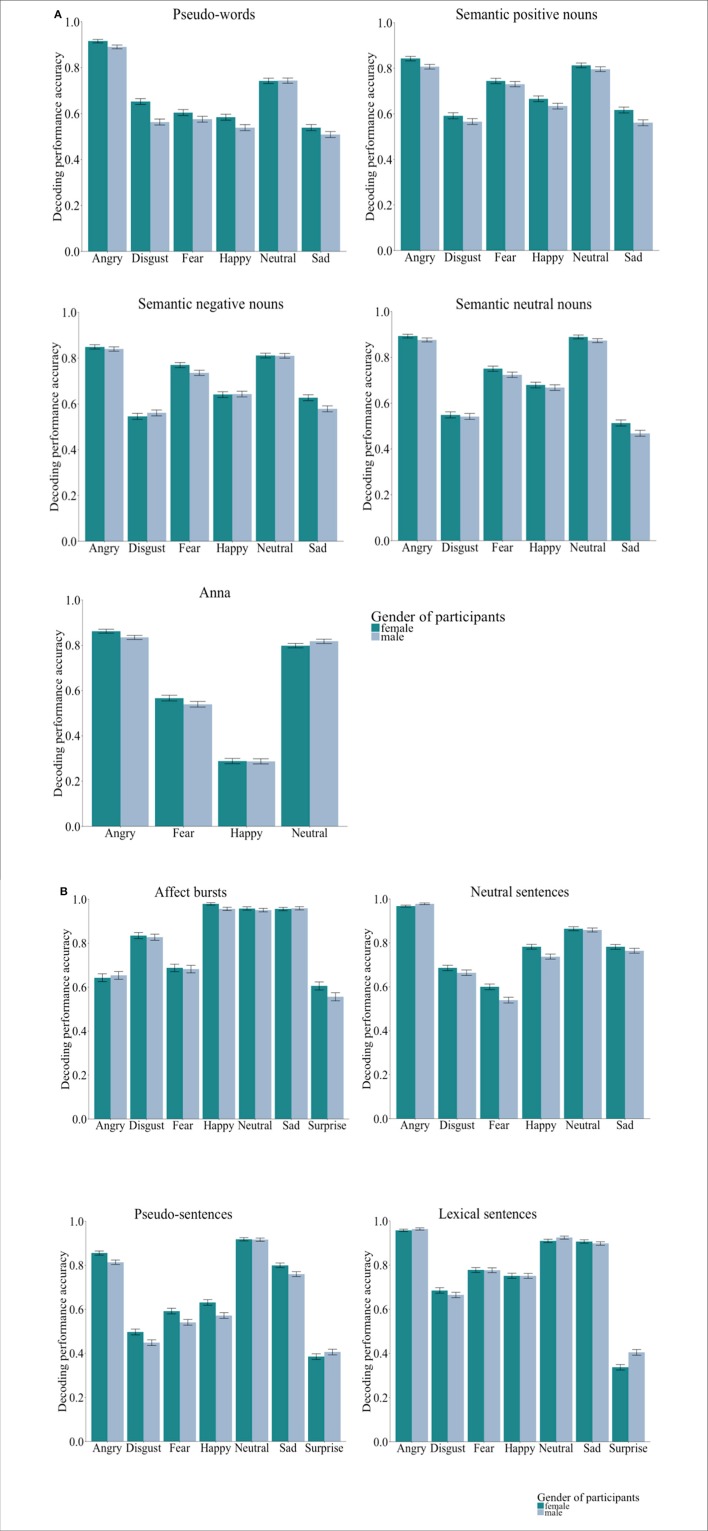
**(A)** *Group words* (*n* = 145, 71 females). Bar charts showing the performance accuracy by listeners' gender. Error bars represent the standard error. As it can be observed, for the majority of emotion categories by databases, females had higher decoding performance accuracy than males. **(B)** *Group sentences* (*n* = 145, 72 females). Bar charts showing the performance accuracy by listeners' gender. Error bars represent the standard error. As it can be observed, for the majority of emotion categories by databases, females had higher decoding performance accuracy than males.

The quasi-binomial logistic models showed significant first order interactions between listeners' gender and emotions for both groups [*Group Words*: $\chi_{\left(5\right)}^{\text{2}}$ = 26.60, *p* < 0.001; *Group Sentences*: $\chi_{\left(6\right)}^{\text{2}}$ = 19.60, *p* = 0.029]. When testing the performance accuracy by stimulus type there were no significant interactions between listeners' gender and emotions [*Anna*: $\chi_{\left(3\right)}^{\text{2}}$ = 7.61, *p* = 0.644; *pseudo-words*: $\chi_{\left(5\right)}^{\text{2}}$ = 15.18, *p* = 1.00; *semantic positive nouns:* $\chi_{\left(5\right)}^{\text{2}}$ = 6.73, *p* = 1.00; *semantic negative nouns:* $\chi_{\left(5\right)}^{\text{2}}$ = 14.96, *p* = 0.124; *semantic neutral nouns*: $\chi_{\left(5\right)}^{\text{2}}$ = 3.12, *p* = 1.00; *pseudo-sentences*: $\chi_{\left(6\right)}^{\text{2}}$ = 17.36, *p* = 0.075; *lexical sentences*: $\chi_{\left(6\right)}^{\text{2}}$ = 9.60, *p* = 1.00; *neutral sentences*: $\chi_{\left(5\right)}^{\text{2}}$ = 4.05, *p* = 1.00; *affect bursts*: $\chi_{\left(6\right)}^{\text{2}}$ = 5.90, *p* = 0.848]. Figures 1A,B illustrate the performance accuracy by listeners' gender and emotion categories, separated for both, *Group-Words* and *Group-Sentences*.

To describe the size of the difference between females and males when decoding emotions, a *Wilcoxon Mann-Whitney* test was implemented. Results showed that overall females (*M* = 0.67, *SD* = 0.06) were significantly better than males (*M* = 0.64, *SD* = 0.06) at decoding emotions from pseudo-sentences, *z* = 2.87, *p* = 0.033, *d* = 0.49. No significant differences between females and males by emotion category were observed, with effect sizes close to zero (*d* ≤ 0.10) or in the small (0.11 < *d* < 0.35) range. Overall, the results indicated the existence of a small decoding effect favoring females across all emotions (0.15 < *d* < 0.34) and stimulus types (*d* = 0.31). The parameter estimates by listeners' gender for each emotion category, across all emotions and stimulus types are presented in Tables 4A,B.

**Table 4A:** Group words: Means, standard deviations, z-scores, *p*-values, and effect sizes of performance accuracy by listeners' gender.

**Stimulus type**	**Emotion category**	**Female**		**Male**				
		***n***	***M (SD)***	***n***	***M (SD)***	***z***	***p***	***d***
Anna	*Angry*	71	0.86 (0.09)	74	0.83 (0.12)	1.26	1.00	0.26
	*Fear*	71	0.57 (0.16)	74	0.54 (0.15)	1.03	1.00	0.17
	*Happy*	71	0.29 (0.11)	74	0.29 (0.11)	−0.23	1.00	0.01
	*Neutral*	71	0.80 (0.16)	74	0.82 (0.14)	−0.43	1.00	0.12
	Overall	71	0.63 (0.06)	74	0.62 (0.06)	0.82	1.00	0.15
Pseudo-words	*Angry*	71	0.92 (0.09)	74	0.89 (0.12)	1.48	0.967	0.24
	*Disgust*	71	0.65 (0.22)	74	0.56 (0.22)	2.65	0.057	0.41
	*Fear*	71	0.60 (0.19)	74	0.58 (0.19)	0.82	1.00	0.15
	*Happy*	71	0.58 (0.17)	74	0.54 (0.21)	1.27	1.00	0.23
	*Neutral*	71	0.74 (0.16)	74	0.74 (0.20)	−0.50	1.00	0.00
	*Sad*	71	0.54 (0.29)	74	0.51 (0.28)	0.65	1.00	0.11
	*Overall*	71	0.67 (0.11)	74	0.64 (0.12)	1.74	0.572	0.32
Semantic positive nouns	*Angry*	71	0.84 (0.11)	74	0.81 (0.13)	1.69	0.637	0.30
	*Disgust*	71	0.59 (0.22)	74	0.57 (0.23)	0.67	1.00	0.11
	*Fear*	71	0.74 (0.16)	74	0.73 (0.17)	0.65	1.00	0.08
	*Happy*	71	0.67 (0.14)	74	0.64 (0.15)	1.31	1.00	0.22
	*Neutral*	71	0.81 (0.15)	74	0.80 (0.15)	0.68	1.00	0.11
	*Sad*	71	0.62 (0.26)	74	0.56 (0.27)	1.28	1.00	0.21
	*Overall*	71	0.71 (0.08)	74	0.68 (0.09)	1.96	0.352	0.34
Semantic negative nouns	*Angry*	71	0.85 (0.12)	74	0.84 (0.11)	0.90	1.00	0.08
	*Disgust*	71	0.55 (0.21)	74	0.56 (0.22)	−0.35	1.00	0.07
	*Fear*	71	0.77 (0.13)	74	0.74 (0.15)	1.42	1.00	0.23
	*Happy*	71	0.64 (0.15)	74	0.64 (0.19)	−0.60	1.00	0.01
	*Neutral*	71	0.81 (0.17)	74	0.81 (0.16)	0.18	1.00	0.01
	*Sad*	71	0.63 (0.26)	74	0.59 (0.26)	1.26	1.00	0.19
	Overall	71	0.71 (0.07)	74	0.69 (0.09)	0.62	1.00	0.15
Semantic neutral nouns	*Angry*	71	0.89 (0.10)	74	0.88 (0.10)	1.08	1.00	0.16
	*Disgust*	71	0.55 (0.21)	74	0.54 (0.21)	0.24	1.00	0.03
	*Fear*	71	0.75 (0.17)	74	0.72 (0.18)	1.09	1.00	0.15
	*Happy*	71	0.68 (0.15)	74	0.67 (0.20)	−0.22	1.00	0.06
	*Neutral*	71	0.89 (0.13)	74	0.87 (0.14)	0.43	1.00	0.12
	*Sad*	71	0.51 (0.28)	74	0.47 (0.27)	0.99	1.00	0.16
	Overall	71	0.71 (0.08)	74	0.69 (0.10)	1.17	1.00	0.23
Overall		71	0.69 (0.07)	74	0.67 (0.08)	1.39	0.163	0.31

*The group comparisons between males and females were made using the Wilcoxon-Mann-Whitney test. The decoding performance accuracy was higher for females as indicated by positive z-scores and higher for males as indicated by negative z-scores. The tests were conducted for each emotion separately, across all emotions and stimulus types. All p-values were Bonferroni corrected*.

**Table 4B:** Group Sentences: Means, standard deviations, z-scores, *p*-values and effect sizes of performance accuracy by listeners' gender.

**Stimulus type**	**Emotion category**	**Female**		**Male**				
		***n***	***M (SD)***	***n***	***M (SD)***	***z***	***p***	***d***
Affect bursts	*Angry*	72	0.64 (0.13)	73	0.65 (0.16)	−0.81	1.00	0.07
	*Disgust*	72	0.83 (0.10)	73	0.83 (0.12)	0.23	1.00	0.07
	*Fear*	72	0.69 (0.18)	73	0.68 (0.20)	0.07	1.00	0.03
	*Happy*	72	0.98 (0.05)	73	0.96 (0.11)	0.80	1.00	0.27
	*Neutral*	72	0.96 (0.06)	73	0.95 (0.08)	0.20	1.00	0.11
	*Sad*	72	0.96 (0.09)	73	0.96 (0.08)	0.24	1.00	0.04
	*Surprise*	72	0.61 (0.20)	73	0.56 (0.23)	1.26	1.00	0.23
	*Overall*	72	0.81 (0.05)	73	0.80 (0.06)	0.86	1.00	0.22
Pseudo-sentences	*Angry*	72	0.86 (0.12)	73	0.81 (0.13)	1.92	0.434	0.32
	*Disgust*	72	0.50 (0.17)	73	0.45 (0.17)	1.97	0.393	0.28
	*Fear*	72	0.59 (0.17)	73	0.54 (0.18)	1.83	0.533	0.30
	*Happy*	72	0.63 (0.16)	73	0.57 (0.16)	1.99	0.368	0.37
	*Neutral*	72	0.92 (0.09)	73	0.92 (0.09)	−0.26	1.00	0.02
	*Sad*	72	0.80 (0.13)	73	0.76 (0.15)	1.74	0.649	0.28
	*Surprise*	72	0.39 (0.17)	73	0.41 (0.16)	−0.49	1.00	0.12
	*Overall*	72	0.67 (0.06)	73	0.64 (0.06)	2.87	0.033	0.49
Lexical sentences	*Angry*	72	0.96 (0.05)	73	0.96 (0.06)	−1.19	1.00	0.10
	*Disgust*	72	0.69 (0.20)	73	0.66 (0.16)	0.96	1.00	0.11
	*Fear*	72	0.78 (0.15)	73	0.78 (0.17)	−0.29	1.00	0.01
	*Happy*	72	0.75 (0.16)	73	0.75 (0.17)	−0.20	1.00	0.00
	*Neutral*	72	0.91 (0.09)	73	0.92 (0.08)	−0.73	1.00	0.17
	*Sad*	72	0.91 (0.08)	73	0.90 (0.09)	0.53	1.00	0.10
	*Surprise*	72	0.34 (0.18)	73	0.40 (0.20)	−2.08	0.298	0.35
	*Overall*	72	0.76 (0.07)	73	0.77 (0.06)	−0.69	1.00	0.13
Neutral sentences	*Angry*	72	0.97 (0.05)	73	0.98 (0.03)	−1.29	1.00	0.26
	*Disgust*	72	0.69 (0.14)	73	0.66 (0.17)	0.48	1.00	0.14
	*Fear*	72	0.60 (0.21)	73	0.54 (0.18)	1.81	0.491	0.30
	*Happy*	72	0.78 (0.13)	73	0.74 (0.16)	1.45	1.00	0.31
	*Neutral*	72	0.86 (0.13)	73	0.86 (0.13)	0.35	1.00	0.04
	*Sad*	72	0.78 (0.19)	73	0.76 (0.18)	0.75	1.00	0.09
	*Overall*	72	0.78 (0.08)	73	0.76 (0.08)	1.86	0.442	0.30
Overall		72	0.75 (0.05)	73	0.73 (0.05)	1.60	0.110	0.31

*The group comparisons between males and females were made using the Wilcoxon-Mann-Whitney test. The decoding performance accuracy was higher for females as indicated by positive z-scores and higher for males as indicated by negative z-scores. The tests were conducted for each emotion separately, across all emotions and stimulus types. All p-values were Bonferroni corrected*.

### Performance accuracy of identifying vocal emotions by speakers' gender

The logistic regression models showed significant first order interactions between speaker gender and stimuli types [*Group Words*: $\chi_{\left(4\right)}^{\text{2}}$ = 142.80, *p* < 0.001; *Group Sentences*: $\chi_{\left(3\right)}^{\text{2}}$ = 18.50, *p* = 0.003]. Main effects of speaker gender were observed across all stimuli types [*Group Words*: $\chi_{\left(1\right)}^{\text{2}}$ = 42.30, *p* < 0.001; *Group Sentences*: $\chi_{\left(1\right)}^{\text{2}}$ = 589.40, *p* < 0.001], and following stimulus sub-sets: *Anna* [$\chi_{\left(1\right)}^{\text{2}}$ = 75.13, *p* < 0.001], *pseudo-words* [$\chi_{\left(1\right)}^{\text{2}}$ = 22.26, *p* < 0.001], *semantic negative nouns* [$\chi_{\left(1\right)}^{\text{2}}$ = 71.74, *p* < 0.001], *pseudo-sentences* [$\chi_{\left(1\right)}^{\text{2}}$ = 173.65, *p* < 0.001], *lexical sentences* [$\chi_{\left(1\right)}^{\text{2}}$ = 154.70, *p* < 0.001], and *affect bursts* [$\chi^{}2_{\left(1\right)}$ = 40.24, *p* < 0.001]. No main effects of speaker gender were found for *semantic positive nouns* [$\chi_{\left(1\right)}^{\text{2}}$ = 0.43, *p* = 1.00], *semantic neutral nouns* [$\chi_{\left(1\right)}^{\text{2}}$ = 3.05, *p* = 0.997], and *neutral sentences* [$\chi_{\left(1\right)}^{\text{2}}$ = 0.93, *p* = 1.00].

**Figure 2:**
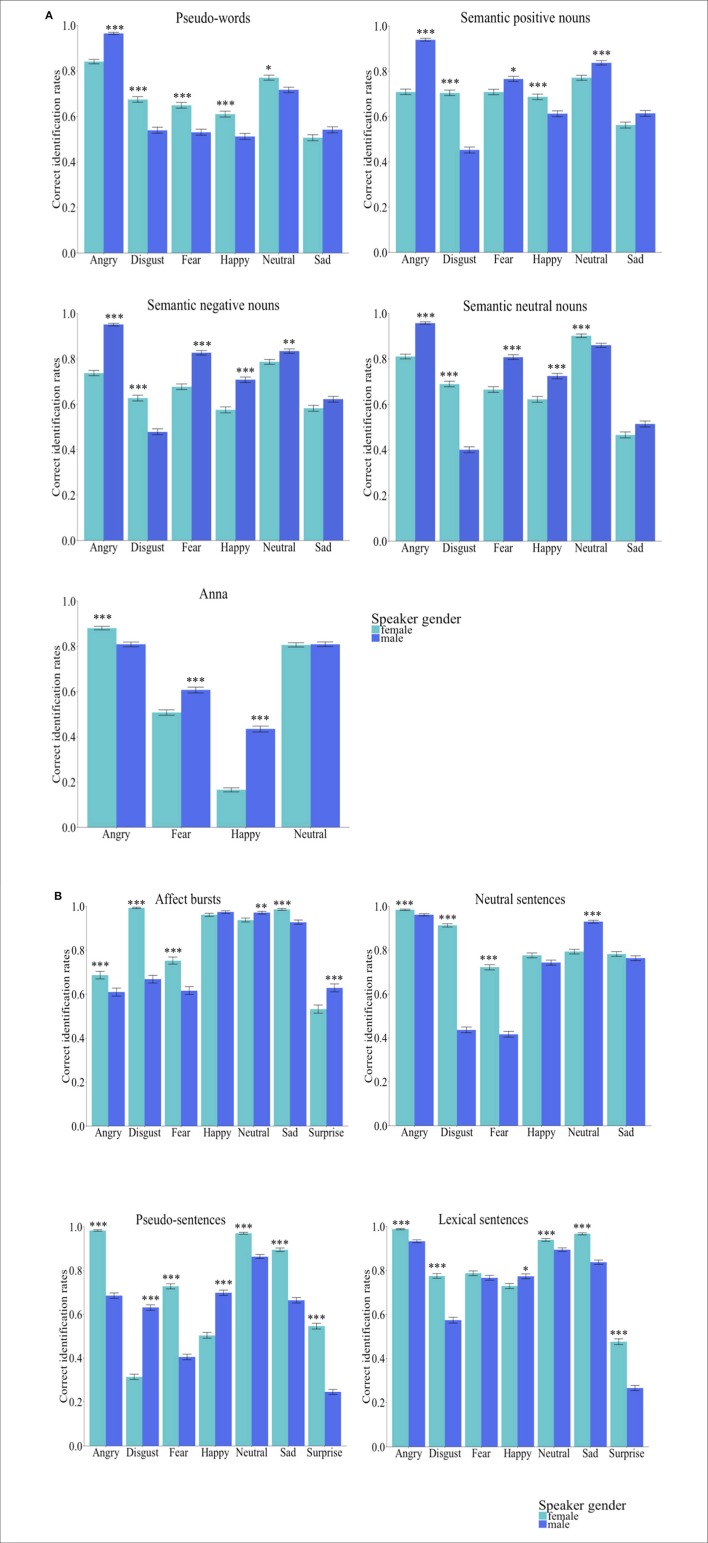
**(A)** *Group words* (*n* = 145, 71 females). Bar charts showing the performance accuracy of identifying emotions by speakers'gender. Error bars represent the standard error. Asterisks mark the significance level: ^*^p < 0.05, ^**^p<*0.01*, ^***^p<*0.001*. As it can be observed, for the majority of emotion categories by databases, the correct identification rates were higher for emotions uttered in a male than a female voice. **(B)** *Group sentences* (*n* = 145, 72 females). Bar charts showing the performance accuracy of identifying emotions by speakers' gender. Error bars represent the standard error. Asterisks mark the significance level: ^*^p < 0.05, ^**^p < 0.01, ^***^p < 0.001. As it can be observed, for the majority of emotion categories by databases, the correct identification rates were higher for emotion uttered in a female than a male voice.

We observed significant first order interactions between speakers' gender and emotions across all stimuli types [*Group Words*: $\chi_{\left(5\right)}^{\text{2}}$ = 842.30, *p* < 0.001; *Group Sentences*: $\chi_{\left(6\right)}^{\text{2}}$ = 726.70, *p* < 0.001] and for each stimulus sub-set [Anna: $\chi_{\left(3\right)}^{\text{2}}$ = 211.41, *p* < 0.001; pseudo-words: $\chi_{\left(5\right)}^{\text{2}}$ = 202.22, *p* < 0.001; semantic positive nouns: $\chi_{\left(5\right)}^{\text{2}}$ = 462.14, *p* < 0.001; semantic negative nouns: $\chi_{\left(5\right)}^{\text{2}}$ = 280.14, *p* < 0.001; semantic neutral nouns: $\chi_{\left(5\right)}^{\text{2}}$ = 465.36, *p* < 0.001; affect bursts:$\chi_{\left(6\right)}^{\text{2}}$ = 243.28, *p* < 0.001; pseudo-sentences: $\chi_{\left(6\right)}^{\text{2}}$ = 1276.99, *p* < 0.001; lexical sentences: $\chi_{\left(6\right)}^{\text{2}}$ = 194.50, *p* < 0.001; neutral sentences: $\chi_{\left(5\right)}^{\text{2}}$ = 449.41, *p* < 0.001]. Figures 2A, B display listeners' performance accuracy when identifying emotions from females' and males' voice.

To analyze whether specific emotions will have higher identification rates when spoken by a female than by a male encoder or vice-versa, a *Wilcoxon-rank-sum* test was fitted. Results showed that, except pseudo-sentences, in all other types of stimuli *disgust* was significantly better identified when uttered by a female than by a male (*p's* < 0.001, 0.42 < *d* < 2.39). In *Group Words*, except for the name Anna, *angry* had higher identification rates in males' than females' voice (*p's* < 0.001, 0.89 < *d* < 1.29), whereas in *Group Sentences* this emotion was better identified when spoken by a female than by a male (*p's* < 0.001, 0.32 < *d* < 1.21). For the other emotion categories, the pattern of results was not as clear-cut: in some types of stimuli utterances were significantly better identified when spoken by female than male actors and vice-versa. Across all emotions, *Anna* (*p* < 0.001, *d* = 0.84) and *semantic negative nouns* (*p* < 0.001, *d* = 0.84) were better identified in the male voice, whereas *pseudo-words* were better identified in the female voice. No significant differences in performance accuracy when male or female actors expressed the emotions were observed for *semantic positive nouns* (*p* = 1.00, *d* = 0.15) and *semantic neutral nouns* (*p* = 0.578, *d* = 0.18). In *Group Sentences*, however, female utterances were significantly better identified than those spoken by male actors (*p's* < 0.001, 0.80 < *d* < 1.62). Across all stimuli types, in *Group Words* vocal expressions had higher identification rates for male actors' expressions of emotion (*p* < 0.001, *d* = 0.40), whereas in *Group Sentences* these were better identified in the female voice (*p* < 0.001, *d* = 2.29). The performance accuracy by speakers' gender for each emotion category, across all emotions and stimulus types is presented in Tables 5A,B.

**Table 5A:** Group Words: Means, standard deviations, z-scores, *p*-values, and effect sizes of identification rates by speakers' gender.

**Stimulus type**	**Emotion category**	**Female**		**Male**					
**Anna**		***n***	***M***	***n***	***M***	***SD (Δ)***	***z***	***p***	***d***
	*Fear*	71	0.51	74	0.61	0.18	−6.01	<0.001	0.57
	*Happy*	71	0.17	74	0.43	0.20	−9.72	<0.001	1.32
	*Angry*	71	0.88	74	0.80	0.15	−5.41	<0.001	0.48
	*Neutral*	71	0.81	74	0.81	0.17	−0.45	1.00	0.01
	*Overall*	71	0.59	74	0.67	0.09	−7.97	<0.001	0.84
Pseudo-words	*Fear*	71	0.65	74	0.53	0.25	5.21	<0.001	0.47
	*Happy*	71	0.61	74	0.51	0.19	5.63	<0.001	0.51
	*Sad*	71	0.51	74	0.54	0.28	−1.54	0.873	0.12
	*Angry*	71	0.84	74	0.96	0.13	−9.23	<0.001	0.99
	*Disgust*	71	0.67	74	0.54	0.32	4.73	<0.001	0.42
	*Neutral*	71	0.78	74	0.72	0.21	3.10	0.013	0.26
	*Overall*	71	0.68	74	0.63	0.09	4.95	<0.001	0.44
Semantic positive nouns	*Fear*	71	0.71	74	0.77	0.21	−3.10	0.013	0.28
	*Happy*	71	0.69	74	0.61	0.23	3.89	<0.001	0.32
	*Sad*	71	0.56	74	0.61	0.32	−2.25	0.170	0.16
	*Angry*	71	0.71	74	0.94	0.18	−10.08	<0.001	1.29
	*Disgust*	71	0.70	74	0.45	0.27	−8.65	<0.001	0.93
	*Neutral*	71	0.77	74	0.84	0.21	−4.60	<0.001	0.32
	*Overall*	71	0.69	74	0.70	0.09	−1.51	1.00	0.15
Semantic negative nouns	*Fear*	71	0.68	74	0.83	0.22	−6.92	<0.001	0.68
	*Happy*	71	0.58	74	0.71	0.21	−6.56	<0.001	0.63
	*Sad*	71	0.58	74	0.62	0.30	−1.95	0.362	0.13
	*Angry*	71	0.74	74	0.95	0.19	9.74	<0.001	1.41
	*Disgust*	71	0.63	74	0.48	0.29	5.50	<0.001	0.51
	*Neutral*	71	0.79	74	0.83	0.18	−3.75	0.001	0.26
	*Overall*	71	0.66	74	0.74	0.09	−7.72	<0.001	0.78
Semantic neutral nouns	*Fear*	71	0.67	74	0.81	0.26	−5.80	<0.001	0.55
	*Happy*	71	0.62	74	0.72	0.24	−4.82	<0.001	0.43
	*Sad*	71	0.47	74	0.51	0.30	−2.14	0.227	0.16
	*Angry*	71	0.81	74	0.96	0.16	−8.82	<0.001	0.89
	*Disgust*	71	0.69	74	0.40	0.29	8.53	<0.001	1.00
	*Neutral*	71	0.90	74	0.86	0.15	3.82	<0.001	0.27
	*Overall*	71	0.69	74	0.71	0.10	−1.74	0.578	0.18
Overall		71	0.67	74	0.69	0.07	−4.51	<0.001	0.40

*The group comparisons between males and females were made using the Wilcoxon-rank-sum test. SD(Δ) = standard deviation of the difference of relative frequencies female and relative frequencies male. Positive z-scores indicate that the emotional portrayals had higher identification rates when spoken by female actors', whereas negative z-scores denote that the performance was higher when the emotions were spoken by male actors'. The tests were conducted for each emotion separately, across all emotions and stimulus types. All p-values were Bonferroni corrected*.

**Table 5B:** Group Sentences: Means, standard deviations, z-scores, p-values and effect sizes of identification rates by speakers' gender.

**Stimulus type**	**Emotion category**	**Female**		**Male**					
		***n***	***M***	***n***	***M***	***SD(Δ)***	***z***	***p***	***d***
Affect bursts	*Fear*	72	0.75	73	0.62	0.28	5.25	<0.001	0.48
	*Happy*	72	0.97	73	0.96	0.19	−1.09	1.00	0.12
	*Sad*	72	0.99	73	0.93	0.12	5.41	<0.001	0.48
	*Angry*	72	0.69	73	0.61	0.24	4.52	<0.001	0.32
	*Disgust*	72	0.99	73	0.68	0.22	10.28	<0.001	1.51
	*Neutral*	72	0.97	73	0.94	0.13	3.24	0.009	0.26
	*Surprise*	72	0.53	73	0.63	0.26	−3.96	<0.001	0.37
	*Overall*	72	0.84	73	0.77	0.09	8.10	<0.001	0.80
Pseudo-sentences	*Fear*	72	0.73	73	0.40	0.27	9.47	<0.001	1.18
	*Happy*	72	0.50	73	0.70	0.23	−8.10	<0.001	0.86
	*Sad*	72	0.90	73	0.66	0.25	8.50	<0.001	0.93
	*Angry*	72	0.98	73	0.69	0.25	10.21	<0.001	1.21
	*Disgust*	72	0.31	73	0.63	0.22	−9.76	<0.001	1.42
	*Neutral*	72	0.97	73	0.86	0.12	8.60	<0.001	0.89
	*Surprise*	72	0.55	73	0.25	0.25	9.43	<0.001	1.19
	*Overall*	72	0.71	73	0.60	0.09	9.49	<0.001	1.22
Lexical sentences	*Fear*	72	0.79	73	0.77	0.22	0.72	1.00	0.10
	*Happy*	72	0.73	73	0.77	0.18	−2.91	0.029	0.25
	*Sad*	72	0.97	73	0.84	0.17	7.97	<0.001	0.77
	*Angry*	72	0.99	73	0.93	0.09	7.18	<0.001	0.64
	*Disgust*	72	0.78	73	0.57	0.25	7.90	<0.001	0.82
	*Neutral*	72	0.94	73	0.90	0.13	4.46	<0.001	0.33
	*Surprise*	72	0.48	73	0.27	0.19	9.10	<0.001	1.10
	*Overall*	72	0.80	73	0.72	0.07	9.78	<0.001	1.20
Neutral sentences	*Fear*	72	0.72	73	0.42	0.20	10.09	<0.001	1.48
	*Happy*	72	0.78	73	0.74	0.17	2.36	0.128	0.19
	*Sad*	72	0.78	73	0.76	0.23	0.21	1.00	0.08
	*Angry*	72	0.98	73	0.96	0.07	3.88	<0.001	0.33
	*Disgust*	72	0.91	73	0.44	0.20	10.47	<0.001	2.39
	*Neutral*	72	0.93	73	0.79	0.17	8.31	<0.001	0.80
	*Overall*	72	0.83	73	0.71	0.07	10.23	<0.001	1.62
Overall		72	0.79	73	0.69	0.04	10.48	<0.001	2.29

*The group comparisons between males and females were made using the Wilcoxon-rank-sum test. SD(Δ) = standard deviation of the difference of relative frequencies female and relative frequencies male. Positive z-scores indicate that the emotional portrayals had higher identification rates when spoken by female actors', whereas negative z-scores denote that the performance was higher when the emotions were spoken by male actors'. The tests were conducted for each emotion separately, across all emotions and stimulus types. All p-values were Bonferroni corrected*.

### Interplay of decoder and encoder gender and emotion

**Figure 3:**
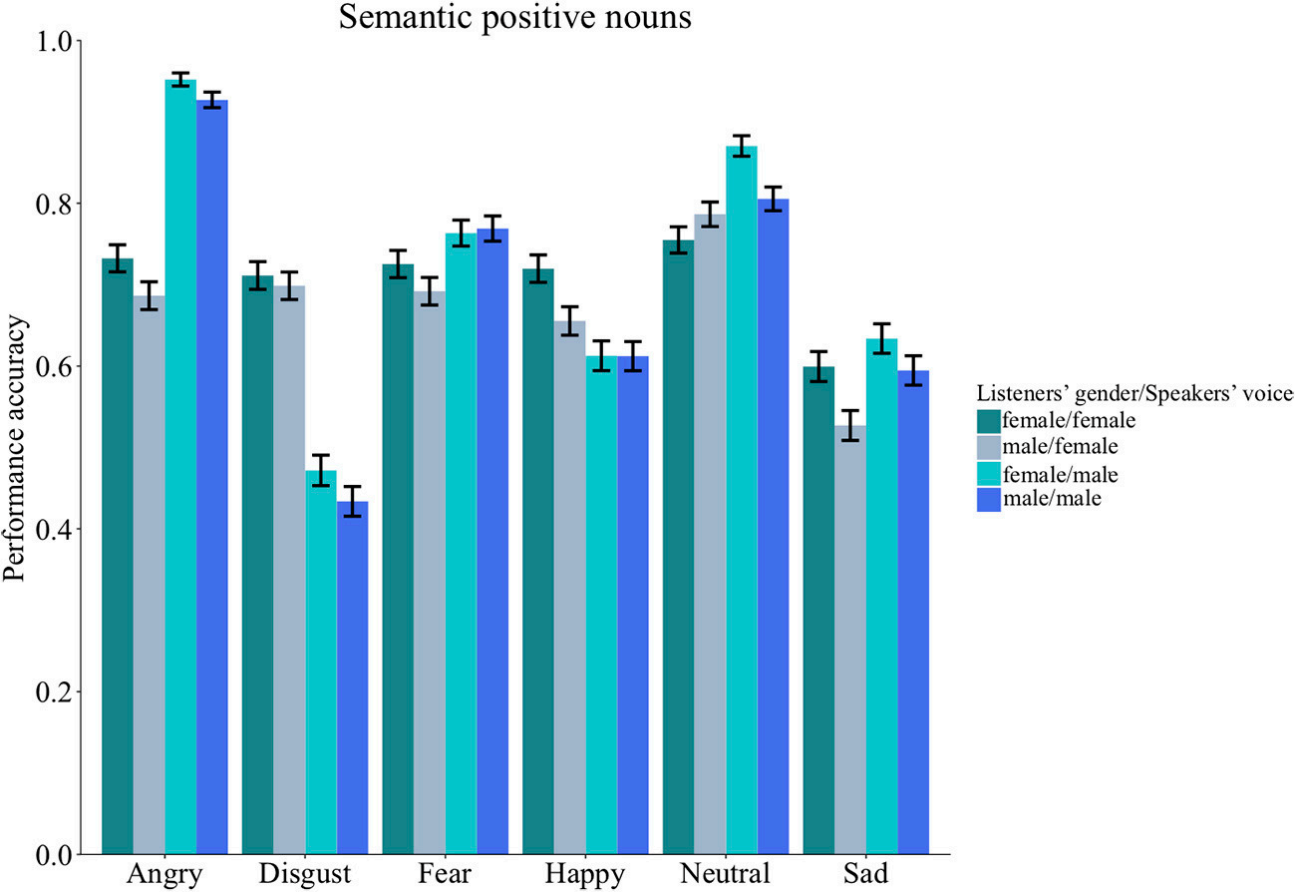
*Group words* (*n* = 145, 71 females). Bar charts showing the performance accuracy listeners' gender, speakers' gender and emotion categories. Error bars represent the standard error. As it can be observed, the second order interaction pattern is explained by the inspection of the average ratings showing different gender patterns conditional on emotion categories. For instance, female listeners had higher recognition accuracy for the emotion category happiness encoded in the female voice and higher recognition accuracy for the neutral category encoded in the male voice. In contrast, male listeners had lower recognition accuracy for the emotion category sad when spoken by a female.

No interactions between listeners and speakers gender was found across stimuli types [*Group Words*: $\chi_{\left(1\right)}^{\text{2}}$ = 0.30, *p* = 1.00; *Group Sentences*: $\chi_{\left(1\right)}^{\text{2}}$ = 0.10, *p* = 1.00] or for any of the stimuli sub-sets [Anna: $\chi_{\left(1\right)}^{\text{2}}$ = 1.41, *p* = 1.00; pseudo-words: $\chi_{\left(1\right)}^{\text{2}}$ = 0.85, *p* = 1.00; semantic positive nouns: $\chi_{\left(1\right)}^{\text{2}}$ = 0.06, *p* = 1.00; semantic negative nouns: $\chi_{\left(1\right)}^{\text{2}}$ = 0.25, *p* = 1.00; semantic neutral nouns: $\chi_{\left(1\right)}^{\text{2}}$ = 2.80, *p* = 1.00; affect bursts: $\chi_{\left(1\right)}^{\text{2}}$ = 0.21, *p* = 1.00; pseudo-sentences: $\chi_{\left(1\right)}^{\text{2}}$ = 0.31, *p* = 1.00; lexical sentences: $\chi_{\left(1\right)}^{\text{2}}$ = 0.20, *p* = 1.00; neutral sentences: $\chi_{\left(1\right)}^{\text{2}}$ = 1.67, *p* = 1.00]. The quasi-binomial logistic regression model revealed a significant second order interaction between speaker gender (encoder), listener gender (decoder), and emotion for semantic positive nouns [$\chi_{\left(5\right)}^{\text{2}}$ = 17.94, *p* = 0.044]. This second order interaction pattern is explained by the inspection of the average ratings showing different gender patterns conditional on emotion categories (see Figure 3).

No second order interactions were found across stimuli types [*Group Words*: $\chi_{\left(5\right)}^{\text{2}}$ = 15.00, *p* = 0.164; *Group Sentences*: $\chi_{\left(6\right)}^{\text{2}}$ = 4.50, *p* = 1.00] and for any of the other stimuli sub-sets: Anna [$\chi_{\left(3\right)}^{\text{2}}$ = 2.57, *p* = 1.00], pseudo-words [$\chi_{\left(5\right)}^{\text{2}}$ = 10.04, *p* = 0.927], semantic negative nouns [$\chi_{\left(5\right)}^{\text{2}}$ = 6.63, *p* = 1.00], semantic neutral nouns [$\chi_{\left(5\right)}^{\text{2}}$ = 2.36, *p* = 1.00], affect bursts [$\chi_{\left(6\right)}^{\text{2}}$ = 4.94, *p* = 1.00], pseudo-sentences [$\chi_{\left(6\right)}^{\text{2}}$ = 5.64, *p* = 1.00], lexical sentences [$\chi_{\left(6\right)}^{\text{2}}$ = 5.70, *p* = 1.00] and neutral sentences [$\chi_{\left(5\right)}^{\text{2}}$ = 2.01, *p* = 1.00].

## Discussion

The present study aimed at investigating gender differences in the recognition of vocal emotions. Specifically, we investigated any gender-specific advantage for the decoding of vocal emotions that were presented across a variety of stimulus types and emotion categories. A second objective was to assess whether the speakers' gender impacts on identification accuracy for different types of vocal emotions. Finally, we explored potential interactions between listeners' and speakers' gender for the identification of vocal emotions. The stimuli used in this study included a wide range of vocal utterances (e.g., words/pseudo-words, sentences/pseudo-sentences, affect bursts) that were expressing different emotions [i.e., anger, disgust, happy, fear, sadness, and surprise, or no emotion (neutral)]. These characteristics of the stimulus set allowed us to assess gender differences for the recognition of vocal emotions in a differentiated manner and to provide parameter estimates on the magnitude of these effects. Especially the latter represents a largely neglected aspect within the vocal emotion literature.

Overall, our results showed that in each of the databases there were large differences in the recognition rates between emotions confirming well-established findings that recognition accuracy depends largely on the emotion category concerned (e.g., Scherer et al., 2001). Furthermore, we observed that performance accuracy is modulated by listeners' and speakers' gender and can significantly vary across stimulus types and emotion categories. Finally, we found that speaker gender had a significant impact on how listeners judged specific emotions from the voice. These findings will be discussed in detail in the following sub-sections.

### Performance accuracy by listeners' gender

We observed a significant main effect of gender reflecting that females outperformed males at categorizing emotions in vocal stimuli. The direction of this effect is consistent with previous findings on the recognition of non-verbal expressions of emotion (e.g., Hall, 1978; Collignon et al., 2010; Kret and de Gelder, 2012; Thompson and Voyer, 2014; Wingenbach et al., 2018) and emotional prosody in particular (e.g., Bonebright et al., 1996; Scherer et al., 2001; Paulmann and Uskul, 2014; Keshtiari and Kuhlmann, 2016).

An interesting pattern observed in our study is that females outperformed males when listening to emotionally produced pseudo-speech. Although the differences between females and males were not significant for emotion-specific categories the results clearly showed that overall females outperformed males when recognizing emotions from pseudo-sentences and had slightly higher recognition rates when decoding emotions from pseudo-words. As pseudo-speech lacks semantic meaning, one possible explanation for this effect is that women compared to men are better decoders under conditions of minimal stimulus information [see for example, *child-rearing hypothesis* (Babchuk et al., 1985) according to which females due to their role as primary caretakers have developed “evolved adaptions” hypothesized to include the fast and accurate decoding especially, for negative emotions]. However, the female advantage was significant only for pseudo-sentences and, thus until further evidence is provided on the robustness of these effects, this interpretation should be approached with caution. One example related to this interpretation is a large-sample study (*N* = 5,872) conducted by Fischer et al. (2018) that failed to replicate earlier findings assuming that females are better than males when categorizing discrete emotions in faces under situations of minimal stimulus information. Previous studies revealed that females score higher than males in decoding specific emotions such as happiness, fear, sadness or neutral expressions and that both genders perform equally well for emotions spoken in an angry and disgusted tone of voice (for an overview see Table 1). Our results partially support these findings. On the one hand, we were able to show that the performance accuracy between females and males did not differ for emotions spoken in an angry and disgusted tone of voice. The absence of a gender specific advantage for decoding a socially salient vocal emotion such as anger may be because humans (and other primates) are biologically prepared or “hard-wired” (Öhman, 1993) to respond rapidly to specific stimuli (e.g., screams; alarm calls) in the environment, independent of gender. Moreover, it has been suggested that anger and disgust are expressions that signal the rejection of something or someone (Schirmer, 2013) and, thus, one could argue that they place an equal demand on attentional resources regardless of gender. On the other hand, we found no evidence that females outperform males when decoding distressing (i.e., sad, fear), happy and neutral emotions from the voice. Similar to other findings on gender differences in emotion recognition (e.g., Fischer et al., 2018), the magnitude between genders we observed for the decoding of vocal emotions was relatively small. In our study, however, the direction of this effect consistently showed a female advantage for these specific emotions. Incorporating this pattern in biological and socialization models, one could assume that due to their ascribed nurturing, affiliative, and less dominant role (see Schirmer, 2013, for a review; Hess et al., 2005) women might have developed a higher sensitivity to minimal affective signals (e.g., recognition of infants' fleeting and subtle emotional signals) which may contribute to their advantage in understanding other persons' emotional states. Nevertheless, the variety of methodologies used in previous research posits difficulties when aiming to draw a conclusion against or in favor of an “female advantage” toward these specific emotions. Although our results partially suggest that females may have an advantage when decoding emotions from the voice, these effects might relate to the specific stimulus sets rather than female sensitivity toward particular emotions. It seems plausible that this finding is attributable to the different number of emotional categories included in the stimulus sets. For instance, some stimulus sets covered less emotional categories (e.g., Anna) than the options participants were offered to choose from. Offering emotional categories not included in the stimulus set, could lead to a systematic error in the face of a dichotomous choice between an emotion included in the set and one not included (e.g., happy vs. surprise). Another possible explanation could be a bias toward “negative” emotions, due to the majority of emotional categories being negative (four out of seven options). In addition, research has shown that speakers' pitch contour largely depends on the type and length of stimuli (e.g., words vs. sentences; see Jürgens et al., 2015, for a discussion) and, thus, one could argue that the results on emotion specific effects were affected by the acoustic properties of stimuli (e.g., pitch, timing, voice quality; see Banse and Scherer, 1996; Juslin and Laukka, 2003, for details), which might have varied between the stimulus sets.

### Performance accuracy of identifying vocal emotions by speakers' gender

Despite observing some variability between genders in the expression of emotions for certain types of stimuli (e.g., *Anna* and *nouns* with a semantic positive and negative connotation were better identified when spoken by males), overall performance accuracy was significantly higher when females expressed the emotions. While findings for emotion-specific categories are pretty much inconsistent across studies, our results showed significantly higher identification rates for disgust when spoken by a female. However, for the other emotions categories, this pattern was less straightforward than one would expect. For instance, the identification rates for portrayals of anger were not consistently higher when spoken by a male. Likewise, happy, fearful or sad tone of voice were not invariably better identified when the speaker was a female (see Table 1 for an overview on previous findings). Enhanced identification of women compared to men's emotional expressions has been shown in both, facial (e.g., Hall, 1984) and vocal domains (e.g., Scherer et al., 2001; Belin et al., 2008). Previous reviews addressing gender-related patterns for the expression of emotions have suggested that these predispositions emerge as a result of various factors ranging from biologically innate traits, social norms and skills to situational contexts (e.g., see Chaplin, 2015; Fischer and LaFrance, 2015 for an overview). While the overall female advantage in the expression of emotions is advocated across studies on non-verbal communication (Hall, 1984), less clear is the evidence on why females and males differ in how well they can express particular emotions.

In our study, the mixed pattern for emotion-specific effects indicates that in the vocal domain, the reliability of emotion judgments is not systematically influenced by encoders' gender and the related stereotypes of emotional expressivity. Prior studies suggested that encoders' success in the speech channel may vary with the standardized utterance used (Banse and Scherer, 1996; Juslin and Laukka, 2003). As our stimulus sets were standardized utterances selected from validated databases we cannot clearly comment on similarities within or differences between the stimulus sets that might explain the observed mixed-pattern of results. One can assume, however, that in each database the instructions given to encoders' when portraying the emotions were different. This might have increased the chance that encoders differentially produced high- and low-intensity variants of vocal expressions of emotion and, thus one could speculate that, independent of gender, stimuli with higher-intensity were better identified than those with low intensity (Banse and Scherer, 1996; Juslin and Laukka, 2001). Another potential explanation for these variations in performance accuracy is, that in all databases the emotional expressions were recorded in a controlled setting through professional and non-professional actors. They were thus not real-life emotional expressions. While the methods of emotion simulation offer high experimental control, the validity of prosodic stimuli derived from these measures is limited (Scherer, 1986) and may boost *recognition accuracy* (Sauter and Fischer, 2017). Previous studies found that speakers often portray stereotypes of emotions and might differ in the quality of their emotional portrayals (e.g., one speaker might be very good at portraying happiness but not fear, whereas another speaker's performance might show the opposite pattern; Scherer, 1986; Banse and Scherer, 1996). More recent studies complement this evidence by showing that speakers with less acting experience might encounter difficulties when asked, for instance, to emote in a language devoid of meaning (e.g., Paulmann et al., 2016). Similarly, past work has shown that emotion categories sharing the same dimension of valence (e.g., happiness and surprise) and arousal (e.g., anger and fear) are more likely to be confused (e.g., Banse and Scherer, 1996). Thus, it is plausible that enacted emotions, expressed in isolation (i.e., without situational context) and belonging to the same valence category, might have challenged not only encoders' but also listeners' performance accuracy, thereby leading to ambiguous results. Finally, one could argue that the observed patterns in our results with regard to the identification accuracy of particular emotions from speakers' voice might not only be related to above-mentioned characteristics of our selected databases (e.g., types of stimuli, speakers acting experience, context) but they may also be reflected in the similarities and differences of acoustic and spectral profiles of emotional inflections in spoken language and non-verbal vocalizations (see, Banse and Scherer, 1996; Juslin and Laukka, 2001; Sauter et al., 2010, for details), which can be independent of encoders' gender.

### Interplay between listeners, speakers gender and emotion categories

In contrast to previous findings (e.g., Bonebright et al., 1996; Belin et al., 2008) an interesting pattern we observed in our study is related to the significant interaction between listeners' gender, speakers' gender and emotions for sematic positive nouns. This showed that females were more sensitive to happy expressions spoken by a female, while sensitivity increased for angry, neutral, disgust, and sad expressions when spoken by a male. Although recognition accuracy seems to be contingent on the emotion being decoded as well as the speaker's gender, it is not clear whether the influence of encoder gender on these emotions reflects systematic properties of how these emotions are decoded and labeled, or whether certain artifacts may have been introduced by the semantic category of the stimuli (e.g., positive content spoken in an angry voice). As this pattern was present only for this type of stimuli we do not have a clear explanation for this effect. At most, one could speculate that females might be disposed to display fast and accurate decoding strategies in the face of an apparently conflicting message presented through semantics to detect credible cues about a speaker's true attitude and intentions. Words with a positive and negative semantic connotation, for instance, were found to have a processing advantage over neutral words (e.g., Schacht and Sommer, 2009a,b) and, thus one may speculate that this type of stimuli (here meaningful nouns) that either express an emotional state (e.g., happiness) or elicit one (e.g., shame) provoke differential responses in females and males.

### Strengths, limitations and future research

As emphasized by previous research and corroborated by our data, there are several advantages to control for factors believed to be central when assessing emotion recognition ability. First, the ecological validity of emotion recognition tasks can be expected to increase when a large number of stimuli containing a wider range of emotional expressions is studied. Second, employing gender-balanced samples allows the control of possible main effects in emotion recognition ability while examining potential interaction effects between decoders' and encoders'. Finally, presenting participants with one out of several emotions reduces the likelihood of judges arriving at the correct answer by using exclusion and probability rules. Given that gender differences in the recognition of emotions are generally reported as small or even absent, the present study extends previous findings to show that the female advantage becomes more evident when using a variety of stimuli, a larger number of speakers and a wider range of emotions. Although, we agree to some extent with proponents of gender similarity hypothesis (e.g., Hyde, 2014) that this female advantage should not be over-interpreted, our results clearly indicate that in the vocal domain, there was an underlying consistency toward a female advantage across a wide range of presented stimuli. Therefore, we believe that before under-interpreting these effects, one should consider them within the larger context of the more recent literature (e.g., Wingenbach et al., 2018), which similar to our study, demonstrated that improved methodologies and analysis (e.g., balanced design) help to assess the differences between genders in a more representative and generalizable fashion.

In our study, results showed some strong differences favoring each gender when decoding specific emotions from speakers' voice yet, this pattern was less straightforward than we expected. Although all selected stimuli were from validated databases, the variations within our results may simply reflect inconsistent procedures attributable to database characteristics (e.g., speakers' training, baseline vocal qualities, recording conditions). Moreover, it should be noted that despite using a variety of stimuli the number of speakers for some stimulus types was quite small (e.g., pseudo-words; lexical sentences). This makes it hard to generalize the effects regarding speaker gender to other speech databases. Future research should, thus, control for these factors and, seek to replicate findings on gender differences in the recognition of vocal emotions by using datasets of stimuli that include fully naturalized speech in emotion-related states to further increase ecological validity.

The absence of certain emotional categories within the databases and the fixed alternatives of emotional categories listeners had to choose from, might have led to lower accuracy in performance due to higher levels of cognitive load imposed by the task format. We chose a fixed-choice response format to compare the results with the majority of prior literature. However, this format may be less ecologically valid (Russel, 1994) and thus, it has been suggested that tasks including “other emotion” as a response alternative (Frank and Stennett, 2001), visual analog scales (Young et al., 2017) or open-ended perspective taking (Cassels and Birch, 2014) may prove more sensitive when measuring individuals' ability to recognize emotions. Moreover, our experiment might have been affected by common method variance such as assessment context (i.e., laboratory), item complexity (e.g., the perception of surprise might be interpreted as positive or negative), and mood state (for a comprehensive review, see Podsakoff et al., 2003).

An unexpected finding within our study was the significant age difference between males and females in both groups. Although several studies demonstrated that advancing age is associated with lower accuracy performance in the recognition of vocal emotions (e.g., Paulmann et al., 2008; Lima et al., 2014), our cohort was rather close in age (i.e., the older adults were not as old as populations reported in the literature). Thus, in future studies it would be interesting to clarify whether there is a critical earlier age period for emotional prosody recognition. This could be done, for example, by testing balanced groups of similar ages (e.g., 18–23; 24–29; 30–35) in order to specify a point of time at which emotional prosody recognition might start to decline with age.

Moreover, it has been suggested that prosodic acoustic parameters (e.g., speech melody, loudness), among other cues (e.g., semantics), provide listeners with a general understanding of the intended emotion and, thus, contribute in a cumulative fashion to the communication and recognition of emotions (Thompson and Balkwill, 2006, 2009). Future studies could explore how much of the variance in recognition rates is explained by similarities or differences in the acoustic attributes of emotive speech and assess the extent to which listeners use these acoustic parameters as a perceptual cue for identifying the portrayed emotion.

The present findings help to establish whether recognition accuracy differs according to listeners' and speakers' gender. Thus, an important step for future research will be to evaluate theories regarding *why* these differences or similarities may occur by taking into account evolutionary, cognitive-learning, socio-cultural, and expectancy-value theories (Hyde, 2014).

Previous research suggested that the visual-modality conveys higher degrees of positivity-negativity, whereas the voice incorporates higher degrees of dominance-submission (e.g., Hall, 1984). Thus, one interesting line of future investigation could explore whether females specialize in visual and males in vocal communication. Finally, as the present study evidenced some differences in emotion decoding and encoding in the auditory modality, it would be worthwhile to investigate how these differences relate to audio-visual integration of emotional signals among men and women. The combination of recognition data with physiological measures (e.g., peripheral indicators of emotional responses), psychosocial (e.g., personality traits) and demographic variables (e.g., age, education), as well as, self-reported trait measures of emotional intelligence and tests to assess participants' ability for sustained attention during an experiment, could help to assess gender differences in emotion recognition in an even more differentiated manner.

## Conclusion

The present study replicates earlier research findings while controlling for several previously unaddressed confounds. It adds to the literature on gender differences for the recognition of vocal emotions by showing a female advantage in decoding accuracy and by establishing that females' emotional expressions are more accurately identified than those expressed by men. Results explain inconsistencies in the past literature in which findings of female superiority for identifying vocal emotions remain mixed by highlighting that the effect emerges for particular stimulus categories and under controlled environments. The partially mixed pattern of results in the current experimental task should be further investigated in natural settings, to assess whether males and females are attuned toward specific emotions in more realistic contexts.

## Author contributions

AL and AS designed the study and wrote the manuscript. AL conducted the experiment and data analysis. Both authors have given approval for the current version to be submitted and agree to be accountable for all aspects of the work in ensuring that questions related to the accuracy or integrity of any part of the work are appropriately investigated and resolved.

### Conflict of interest statement

The authors declare that the research was conducted in the absence of any commercial or financial relationships that could be construed as a potential conflict of interest.

## Footnotes

^1^We decided to use the term “gender” instead of “sex“ because this concept has a wider connotation and is not purely assigned by genetics. Participants had the option to provide a textual answer with regard to their gender (“Geschlecht”), yet all of them identified either as male or female (none of them wrote “sexless,” “I am born male but feel female” etc.).
