# Supplementary material for: Gender Differences in the Recognition of Vocal Emotions
Source: Front Psychol. 2018 Jun 5;9:882. doi: 10.3389/fpsyg.2018.00882 (PMC5996252; doi:10.3389/fpsyg.2018.00882)
Supplement: Supplementary file 1 [file Data_Sheet_1.docx]

Supplementary Material

**Gender differences in the recognition of vocal emotions**

Adi Lausen^1,2^ & Annekathrin Schacht^1,2^

Adi Lausen*, Annekathrin Schacht

*Correspondence: [adi.lausen@psych.uni-goettingen.de](mailto:adi.lausen@psych.uni-goettingen.de)

| **Table S1 (a) \|** *Global models across all stimuli types for* ***Group Words*** | | | | | |
| --- | --- | --- | --- | --- | --- |
| ***Model terms*** | ***Df*** | ***Deviance*** | ***Resid. Df*** | ***Resid. Dev*** | ***Pr(>Chi)*** |
| NULL |  |  | 82341 | 103392 |  |
| Age | 1 | 3.90 | 82340 | 103328 | 0.838 |
| Confidence | 2 | 4650.90 | 82338 | 98738 | < .001 |
| Speaker gender | 1 | 42.30 | 82337 | 98695 | < .001 |
| Participants gender | 1 | 51.70 | 82336 | 98644 | < .001 |
| Emotions | 5 | 4853.80 | 82331 | 93790 | < .001 |
| Stimuli types | 4 | 826.20 | 82327 | 92964 | < .001 |
| Participants | 142 | 2866.10 | 82185 | 90097 | < .001 |
| Speaker gender x Participants gender | 1 | 0.30 | 82184 | 90097 | 1.00 |
| Age x Stimuli types | 4 | 12.90 | 82180 | 90084 | 0.191 |
| Confidence x Stimuli types | 8 | 89.70 | 82172 | 89995 | < .001 |
| Speaker gender x Stimuli types | 4 | 142.80 | 82168 | 89852 | < .001 |
| Participants gender x Stimuli types | 4 | 16.40 | 82164 | 89835 | 0.038 |
| Emotions x Stimuli types | 18 | 1097.80 | 82146 | 88738 | < .001 |
| Age x Speaker gender | 1 | 9.70 | 82145 | 88728 | 0.030 |
| Age x Emotions | 5 | 78.20 | 82140 | 88650 | < .001 |
| Participants gender x Emotions | 5 | 26.60 | 82135 | 88623 | < .001 |
| Speaker gender x Emotions | 5 | 842.30 | 82130 | 87781 | < .001 |
| Confidence x Participants | 286 | 1010.60 | 81844 | 86770 | < .001 |
| Speaker gender x Participants gender x Emotions | 5 | 15.00 | 81839 | 86755 | 0.164 |
| *Note*: *Resid. Df* = residual degrees of freedom; *Resid. Dev.* = Residual deviance. *P-values* were Bonferroni corrected for multiple testing. | | | | | |

| **Table S1 (b) \|** *Global models across all stimuli types for* ***Group Sentences*** | | | | | |
| --- | --- | --- | --- | --- | --- |
| ***Model terms*** | ***Df*** | ***Deviance*** | ***Resid. Df*** | ***Resid. Dev*** | ***Pr(>Chi)*** |
| NULL |  |  | 68124 | 78330 |  |
| Age | 1 | 13.20 | 68123 | 78317 | 0.003 |
| Confidence | 2 | 5124.9 | 68121 | 73192 | < .001 |
| Speaker gender | 1 | 589.4 | 68120 | 72603 | < .001 |
| Participants gender | 1 | 5.2 | 68119 | 72597 | 0.332 |
| Emotions | 6 | 6956.0 | 68113 | 65641 | < .001 |
| Stimuli types | 3 | 693.8 | 68110 | 64948 | < .001 |
| Participants | 142 | 1344.3 | 67968 | 63603 | < .001 |
| Speaker gender x Participants gender | 1 | 0.1 | 67967 | 63603 | 1.00 |
| Age x Stimuli types | 3 | 1.0 | 67964 | 63602 | 1.00 |
| Confidence x Stimuli types | 6 | 149.2 | 67958 | 63453 | < .001 |
| Speaker gender x Stimuli types | 3 | 18.5 | 67955 | 63435 | 0.003 |
| Participants gender x Stimuli types | 3 | 22.8 | 67952 | 63412 | < .001 |
| Emotions x Stimuli types | 17 | 1990.4 | 67935 | 61421 | < .001 |
| Age x Speaker gender | 1 | 3.7 | 67934 | 61418 | 0.826 |
| Age x Emotions | 6 | 23.4 | 67928 | 61394 | 0.005 |
| Participants gender x Emotions | 6 | 19.6 | 67922 | 61375 | 0.029 |
| Speaker gender x Emotions | 6 | 726.7 | 67916 | 60648 | < .001 |
| Confidence x Participants | 285 | 517.3 | 67631 | 60130 | < .001 |
| Speaker gender x Participants gender x Emotions | 6 | 4.5 | 67625 | 60126 | 1.00 |
| *Note*: *Resid. Df* = residual degrees of freedom; *Resid. Dev.* = Residual deviance. *P-values* were Bonferroni corrected for multiple testing. | | | | | |

| **Table S2 (a) \|** *Quasi-binomial logistic model for* ***Anna*** | | | | | |
| --- | --- | --- | --- | --- | --- |
| ***Model terms*** | ***Df*** | ***Deviance*** | ***Resid. Df*** | ***Resid. Dev*** | ***Pr(>Chi)*** |
| NULL |  |  | 12756 | 16889 |  |
| Age | 1 | 2.97 | 12755 | 16886 | 1.00 |
| Confidence | 2 | 1094.62 | 12753 | 15792 | < .001 |
| Speaker gender | 1 | 75.13 | 12752 | 15717 | < .001 |
| Participants gender | 1 | 0.03 | 12751 | 15717 | 1.00 |
| Emotions | 3 | 2463.87 | 12748 | 13253 | < .001 |
| Participants | 142 | 498.30 | 12606 | 12754 | < .001 |
| Speaker gender x Participants gender | 1 | 1.41 | 12605 | 12753 | 1.00 |
| Age x Speaker gender | 1 | 0.43 | 12604 | 12753 | 1.00 |
| Age x Emotions | 3 | 1.68 | 12601 | 12751 | 1.00 |
| Participants gender x Emotion | 3 | 7.61 | 12598 | 12743 | 0.644 |
| Speaker gender x Emotion | 3 | 211.41 | 12595 | 12532 | < .001 |
| Confidence x Participants | 278 | 364.91 | 12317 | 12167 | < .001 |
| Speaker gender x Participants gender x Emotion | 3 | 2.57 | 12314 | 12164 | 1.00 |
| *Note*: *Resid. Df* = residual degrees of freedom; *Resid. Dev.* = Residual deviance. *P-values* were Bonferroni corrected for multiple testing. | | | | | |

| **Table S2 (b) \|** *Quasi-binomial logistic model for* ***Pseudo-words*** | | | | | |
| --- | --- | --- | --- | --- | --- |
| ***Model terms*** | ***Df*** | ***Deviance*** | ***Resid. Df*** | ***Resid. Dev*** | ***Pr(>Chi)*** |
| NULL |  |  | 17394 | 22420 |  |
| Age | 1 | 8.57 | 17393 | 22411 | 0.042 |
| Confidence | 2 | 959.64 | 17391 | 21452 | < .001 |
| Speaker gender | 1 | 22.26 | 17390 | 21429 | < .001 |
| Participants gender | 1 | 29.18 | 17389 | 21400 | < .001 |
| Emotions | 5 | 1060.19 | 17384 | 20340 | < .001 |
| Participants | 142 | 1072.58 | 17242 | 19267 | < .001 |
| Speaker gender x Participants gender | 1 | 0.85 | 17241 | 19267 | 1.00 |
| Age x Speaker gender | 1 | 1.09 | 17240 | 19266 | 1.00 |
| Age x Emotions | 5 | 14.83 | 17235 | 19251 | 0.136 |
| Participants gender x Emotion | 5 | 15.18 | 17230 | 19236 | 1.00 |
| Speaker gender x Emotion | 5 | 202.22 | 17225 | 19033 | < .001 |
| Confidence x Participants | 275 | 474.52 | 16950 | 18559 | < .001 |
| Speaker gender x Participants gender x Emotion | 5 | 10.04 | 16945 | 18549 | 0.927 |
| *Note*: *Resid. Df* = residual degrees of freedom; *Resid. Dev.* = Residual deviance. *P-values* were Bonferroni corrected for multiple testing. | | | | | |

| **Table S2 (c) \|** *Quasi-binomial logistic model for* ***Semantic positive nouns*** | | | | | |
| --- | --- | --- | --- | --- | --- |
| ***Model terms*** | ***Df*** | ***Deviance*** | ***Resid. Df*** | ***Resid. Dev*** | ***Pr(>Chi)*** |
| NULL |  |  | 17396 | 21343 |  |
| Age | 1 | 0.15 | 17395 | 21343 | 1.00 |
| Confidence | 2 | 816.03 | 17393 | 20527 | < .001 |
| Speaker gender | 1 | 0.43 | 17392 | 20527 | 1.00 |
| Participants gender | 1 | 20.14 | 17391 | 20506 | < .001 |
| Emotions | 5 | 616.96 | 17386 | 19890 | < .001 |
| Participants | 142 | 795.30 | 17244 | 19094 | < .001 |
| Speaker gender x Participants gender | 1 | 0.06 | 17243 | 19094 | 1.00 |
| Age x Speaker gender | 1 | 4.67 | 17242 | 19090 | 0.414 |
| Age x Emotions | 5 | 39.31 | 17237 | 19050 | < .001 |
| Participants gender x Emotion | 5 | 6.73 | 17232 | 19044 | 1.00 |
| Speaker gender x Emotion | 5 | 462.14 | 17227 | 18581 | < .001 |
| Confidence x Participants | 280 | 421.42 | 16947 | 18160 | < .001 |
| Speaker gender x Participants gender x Emotion | 5 | 17.94 | 16942 | 18142 | 0.044 |
| *Note*: *Resid. Df* = residual degrees of freedom; *Resid. Dev.* = Residual deviance. *P-values* were Bonferroni corrected for multiple testing. | | | | | |

| **Table S2 (d) \|** *Quasi-binomial logistic model for* ***Semantic negative nouns*** | | | | | |
| --- | --- | --- | --- | --- | --- |
| ***Model terms*** | ***Df*** | ***Deviance*** | ***Resid. Df*** | ***Resid. Dev*** | ***Pr(>Chi)*** |
| NULL |  |  | 17395 | 21225 |  |
| Age | 1 | 5.24 | 17394 | 21219 | 0.274 |
| Confidence | 2 | 970.85 | 17392 | 20249 | < .001 |
| Speaker gender | 1 | 71.74 | 17391 | 20177 | < .001 |
| Participants gender | 1 | 8.38 | 17390 | 20168 | 0.046 |
| Emotions | 5 | 735.54 | 17385 | 19433 | < .001 |
| Participants | 142 | 815.60 | 17243 | 18617 | < .001 |
| Speaker gender x Participants gender | 1 | 0.25 | 17242 | 18617 | 1.00 |
| Age x Speaker gender | 1 | 2.07 | 17241 | 18615 | 1.00 |
| Age x Emotions | 5 | 21.35 | 17236 | 18594 | 0.008 |
| Participants gender x Emotion | 5 | 14.96 | 17231 | 18579 | 0.124 |
| Speaker gender x Emotion | 5 | 280.14 | 17226 | 18298 | < .001 |
| Confidence x Participants | 280 | 496.20 | 16946 | 17802 | < .001 |
| Speaker gender x Participants gender x Emotion | 5 | 6.63 | 16941 | 17796 | 1.00 |
| *Note*: *Resid. Df* = residual degrees of freedom; *Resid. Dev.* = Residual deviance. *P-values* were Bonferroni corrected for multiple testing. | | | | | |

| **Table S2 (e) \|** *Quasi-binomial logistic model for* ***Semantic neutral nouns*** | | | | | |
| --- | --- | --- | --- | --- | --- |
| ***Model terms*** | ***Df*** | ***Deviance*** | ***Resid. Df*** | ***Resid. Dev*** | ***Pr(>Chi)*** |
| NULL |  |  | 17396 | 21190 |  |
| Age | 1 | 1.07 | 17395 | 21188 | 1.00 |
| Confidence | 2 | 888.61 | 17393 | 20300 | < .001 |
| Speaker gender | 1 | 3.05 | 17392 | 20297 | 0.997 |
| Participants gender | 1 | 9.13 | 17391 | 20288 | 0.029 |
| Emotions | 5 | 1603.56 | 17386 | 18684 | < .001 |
| Participants | 142 | 854.44 | 17244 | 17830 | < .001 |
| Speaker gender x Participants gender | 1 | 2.80 | 17243 | 17827 | 1.00 |
| Age x Speaker gender | 1 | 8.62 | 17242 | 17818 | 0.038 |
| Age x Emotions | 5 | 35.03 | 17237 | 17783 | < .001 |
| Participants gender x Emotion | 5 | 3.12 | 17232 | 17780 | 1.00 |
| Speaker gender x Emotion | 5 | 465.36 | 17227 | 17315 | < .001 |
| Confidence x Participants | 275 | 476.61 | 16952 | 16838 | < .001 |
| Speaker gender x Participants gender x Emotion | 5 | 2.36 | 16947 | 16836 | 1.00 |
| *Note*: *Resid. Df* = residual degrees of freedom; *Resid. Dev.* = Residual deviance. *P-values* were Bonferroni corrected for multiple testing. | | | | | |

| **Table S2 (f) \|** *Quasi-binomial logistic model for* ***Affect bursts*** | | | | | |
| --- | --- | --- | --- | --- | --- |
| ***Model terms*** | ***Df*** | ***Deviance*** | ***Resid. Df*** | ***Resid. Dev*** | ***Pr(>Chi)*** |
| NULL |  |  | 10146 | 10059.1 |  |
| Age | 1 | 0.87 | 10145 | 10058.2 | 1.00 |
| Confidence | 2 | 1116.13 | 10143 | 8942.1 | < .001 |
| Speaker gender | 1 | 40.24 | 10142 | 8901.8 | < .001 |
| Participants gender | 1 | 2.83 | 10141 | 8899.0 | 0.698 |
| Emotions | 6 | 1113.20 | 10135 | 7785.8 | < .001 |
| Participants | 142 | 303.77 | 9993 | 7482.0 | < .001 |
| Speaker gender x Participants gender | 1 | 0.21 | 9992 | 7481.8 | 1.00 |
| Age x Speaker gender | 1 | 1.38 | 9991 | 7480.4 | 1.00 |
| Age x Emotions | 6 | 14.71 | 9985 | 7465.7 | 0.047 |
| Participants gender x Emotion | 6 | 5.90 | 9979 | 7459.8 | 1.00 |
| Speaker gender x Emotion | 6 | 243.28 | 9973 | 7216.5 | < .001 |
| Confidence x Participants | 278 | 362.30 | 9695 | 6854.2 | < .001 |
| Speaker gender x Participants gender x Emotion | 6 | 4.94 | 9689 | 6849.3 | 1.00 |
| *Note*: *Resid. Df* = residual degrees of freedom; *Resid. Dev.* = Residual deviance. *P-values* were Bonferroni corrected for multiple testing. | | | | | |

| **Table S2 (g) \|** *Quasi-binomial logistic model for* ***Pseudo-sentences*** | | | | | |
| --- | --- | --- | --- | --- | --- |
| ***Model terms*** | ***Df*** | ***Deviance*** | ***Resid. Df*** | ***Resid. Dev*** | ***Pr(>Chi)*** |
| NULL |  |  | 20292 | 26212 |  |
| Age | 1 | 2.38 | 20291 | 26209 | 1.00 |
| Confidence | 2 | 1318.73 | 20289 | 24890 | < .001 |
| Speaker gender | 1 | 173.65 | 20288 | 24717 | < .001 |
| Participants gender | 1 | 7.44 | 20287 | 24709 | 0.068 |
| Emotions | 6 | 2784.06 | 20281 | 21925 | < .001 |
| Participants | 142 | 524.65 | 20139 | 21401 | < .001 |
| Speaker gender x Participants gender | 1 | 0.31 | 20138 | 21400 | 1.00 |
| Age x Speaker gender | 1 | 7.21 | 20137 | 21393 | 0.078 |
| Age x Emotions | 6 | 4.62 | 20131 | 21389 | 1.00 |
| Participants gender x Emotion | 6 | 17.36 | 20125 | 21371 | 0.075 |
| Speaker gender x Emotion | 6 | 1276.99 | 20119 | 20094 | < .001 |
| Confidence x Participants | 278 | 347.52 | 19841 | 19747 | 0.005 |
| Speaker gender x Participants gender x Emotion | 5 | 5.64 | 19835 | 19741 | 1.00 |
| *Note*: *Resid. Df* = residual degrees of freedom; *Resid. Dev.* = Residual deviance. *P-values* were Bonferroni corrected for multiple testing. | | | | | |

| **Table S2 (h) \|** *Quasi-binomial logistic model for* ***Lexical sentences*** | | | | | |
| --- | --- | --- | --- | --- | --- |
| ***Model terms*** | ***Df*** | ***Deviance*** | ***Resid. Df*** | ***Resid. Dev*** | ***Pr(>Chi)*** |
| NULL |  |  | 20291 | 22122 |  |
| Age | 1 | 5.80 | 20290 | 22116 | 0.171 |
| Confidence | 2 | 773.30 | 20288 | 21343 | < .001 |
| Speaker gender | 1 | 154.70 | 20287 | 21188 | < .001 |
| Participants gender | 1 | 5.50 | 20286 | 21183 | 0.211 |
| Emotions | 6 | 3745.60 | 20280 | 17437 | < .001 |
| Participants | 142 | 654.00 | 20138 | 16783 | < .001 |
| Speaker gender x Participants gender | 1 | 0.20 | 20137 | 16783 | 1.00 |
| Age x Speaker gender | 1 | 1.50 | 20136 | 16782 | 1.00 |
| Age x Emotions | 6 | 37.50 | 20130 | 16744 | < .001 |
| Participants gender x Emotion | 6 | 9.60 | 20124 | 16735 | 1.00 |
| Speaker gender x Emotion | 6 | 194.50 | 20118 | 16540 | < .001 |
| Confidence x Participants | 279 | 506.70 | 19839 | 16033 | < .001 |
| Speaker gender x Participants gender x Emotion | 6 | 5.70 | 19833 | 16028 | 1.00 |
| *Note*: *Resid. Df* = residual degrees of freedom; *Resid. Dev.* = Residual deviance. *P-values* were Bonferroni corrected for multiple testing. | | | | | |

| **Table S2 (i) \|** *Quasi-binomial logistic model for* ***Neutral sentences*** | | | | | |
| --- | --- | --- | --- | --- | --- |
| ***Model terms*** | ***Df*** | ***Deviance*** | ***Resid. Df*** | ***Resid. Dev*** | ***Pr(>Chi)*** |
| NULL |  |  | 17397 | 20973 |  |
| Age | 1 | 0.08 | 17396 | 20973 | 1.00 |
| Confidence | 2 | 899.54 | 17394 | 20074 | < .001 |
| Speaker gender | 1 | 0.93 | 17393 | 20073 | 1.00 |
| Participants gender | 1 | 5.35 | 17392 | 20067 | 0.243 |
| Emotions | 5 | 1332.93 | 17387 | 18734 | < .001 |
| Participants | 142 | 879.44 | 17245 | 17855 | < .001 |
| Speaker gender x Participants gender | 1 | 1.67 | 17244 | 17853 | 1.00 |
| Age x Speaker gender | 1 | 10.64 | 17243 | 17843 | 0.011 |
| Age x Emotions | 5 | 37.62 | 17238 | 17805 | < .001 |
| Participants gender x Emotion | 5 | 4.05 | 17233 | 17801 | 1.00 |
| Speaker gender x Emotion | 5 | 449.41 | 17228 | 17352 | < .001 |
| Confidence x Participants | 275 | 494.17 | 16953 | 16857 | < .001 |
| Speaker gender x Participants gender x Emotion | 5 | 2.01 | 16948 | 16855 | 1.00 |
| *Note*: *Resid. Df* = residual degrees of freedom; *Resid. Dev.* = Residual deviance. *P-values* were Bonferroni corrected for multiple testing. | | | | | |
